# *In Vivo* RNAi Screen Reveals Neddylation Genes as Novel Regulators of Hedgehog Signaling

**DOI:** 10.1371/journal.pone.0024168

**Published:** 2011-09-08

**Authors:** Juan Du, Junzheng Zhang, Ying Su, Min Liu, Jason K. Ospina, Shengyuan Yang, Alan Jian Zhu

**Affiliations:** 1 School of Life Sciences, Peking University, Beijing, China; 2 Department of Cell Biology, Lerner Research Institute, Cleveland Clinic, Cleveland, Ohio, United States of America; University of Texas MD Anderson Cancer Center, United States of America

## Abstract

Hedgehog (Hh) signaling is highly conserved in all metazoan animals and plays critical roles in many developmental processes. Dysregulation of the Hh signaling cascade has been implicated in many diseases, including cancer. Although key components of the Hh pathway have been identified, significant gaps remain in our understanding of the regulation of individual Hh signaling molecules. Here, we report the identification of novel regulators of the Hh pathway, obtained from an *in vivo* RNA interference (RNAi) screen in *Drosophila*. By selectively targeting critical genes functioning in post-translational modification systems utilizing ubiquitin (Ub) and Ub-like proteins, we identify two novel genes (*dUba3* and *dUbc12*) that negatively regulate Hh signaling activity. We provide *in vivo* and *in vitro* evidence illustrating that dUba3 and dUbc12 are essential components of the neddylation pathway; they function in an enzyme cascade to conjugate the ubiquitin-like NEDD8 modifier to Cullin proteins. Neddylation activates the Cullin-containing ubiquitin ligase complex, which in turn promotes the degradation of Cubitus interruptus (Ci), the downstream transcription factor of the Hh pathway. Our study reveals a conserved molecular mechanism of the neddylation pathway in *Drosophila* and sheds light on the complex post-translational regulations in Hh signaling.

## Introduction

Hedgehog (Hh) signaling is an evolutionarily conserved pathway that governs many crucial developmental events (reviewed in [1], [2]). Dysregulation of the Hh signaling pathway in humans often results in birth defects as well as tumorigenesis in adult organs (reviewed in [3], [4]). Key components of the Hh signaling cascade were initially identified through extensive genetic studies in *Drosophila melanogaster*, among which Hh (the ligand), Patched (Ptc, the receptor), Smoothened (Smo, the activator) and Cubitus interruptus (Ci, the transcription factor) are the most studied. The *hh* gene encodes a secreted protein that triggers a complex cascade of signaling events that are largely conserved from flies to mammals [1–4]. In the absence of Hh ligand, Ptc functions to suppress the activity of Smo. Due to this inhibition, Smo protein is retained in the cytoplasm, where it forms an inhibitory signaling complex with Costal2 (Cos2, a kinesin-like protein), Fused (Fu, a serine/threonine kinase) and Suppressor of Fused (SuFu, a novel regulator). This complex inhibits the activity of the transcription factor Ci by promoting its phosphorylation. Phosphorylated full-length Ci (CiFL, also known as Ci155) is subsequently processed into an N-terminal fragment (CiR, also known as Ci75) through partial degradation of the C-terminal portion of CiFL. CiR, lacking the co-activator binding domain, then moves into the nucleus to repress target gene transcription. In the presence of the Hh ligand, Hh signaling is initiated upon binding of Hh to Ptc, which releases Smo from Ptc inhibition. As a consequence, Smo protein is phosphorylated and relocalizes to the plasma membrane. This leads to dissociation of Ci from the inhibitory signaling complex, thus allowing CiFL to function as a transcription factor to activate the transcription of various Hh target genes ([1–4], and references therein).

Increasing evidence highlights a role of the ubiquitin-proteasome system (UPS) in the regulation of the stability and activity of Ci [5–13]. The majority of cellular protein degradation is subject to the UPS control, in which three different enzyme complexes, in a step-wise fashion, conjugate Ub to specific substrates. E1 (Ub-activating enzyme) and E2 (Ub-conjugating enzyme) are responsible for activating and conjugating Ub proteins, respectively. E3 functions as a Ub protein ligase to transfer Ub protein from the E2 enzyme onto specific substrates. Ubiquitinated substrates are subject to proteolysis in the 26S proteasome, and Ub proteins are recycled from the substrate by the deubiquitinating enzyme (DUB) (reviewed in [14–17]). It is well established that E3 ligases control the substrate specificity in the UPS [16], [17]. Genetic studies in *Drosophila* have identified two distinct E3 ligases for modulating Hh signaling, presumably targeting Ci for cleavage and/or degradation [5–8], [10–12]. Through a poorly understood mechanism, the Slimb (Supernumerary limbs)-Cul1 E3 complex is believed to regulate the activity of CiFL by promoting its partial degradation [5–8]. A second E3 complex, the Rdx (Roadkill)-Cul3 based E3 ligase, was shown to degrade Ci in Hh-responding cells [6], [10–12]. However, whether additional UPS components are involved in the regulation of Ci protein stability remains to be determined. Furthermore, the mechanism by which E3 ligases regulate Ci stability is not known.

Recent studies have revealed various ways in which the activity of these E3 ligase complexes is controlled. One such pathway relies on the covalent attachment of the Ub-like Neural precursor cell Expressed Developmentally Down-regulated protein 8 (NEDD8) to scaffolding Cullin proteins (reviewed in [18]). NEDD8 is conjugated to a conserved C-terminal lysine residue in Cullin proteins through the sequential action of a unique set of E1, E2, and E3 enzymes, a process known as neddylation [18–20]. Neddylated Cullins stimulate the ubiquitination activity of the E3 complex and prevents its association with the inhibitor CAND1 [21]. Neddylated Cullins are also subject to self-ubiquitination and degradation, thus providing a self-regulatory mechanism to maintain a proper level of ubiquitin ligase activity [22].

*Drosophila* wing morphogenesis is one of the most intensively investigated developmental processes for understanding Hh signaling. The stereotypical wing patterning and ample genetic tools make it a favorable system for genetic screens. Several genome-wide screens, using classical forward genetic strategies, have been reported and several novel regulators of the Hh signaling pathway were successfully identified [23–26]. Recently, large-scale *in vitro* RNAi screens have also been performed in cultured fly cells with promising outcomes [27], [28]. However, *in vivo* RNAi screens, aimed at identifying novel Hh signaling regulators, have not been reported. This lack of investigation is in contrast to what has been done for Notch signal transduction [29], [30].

Here, we report an *in vivo* RNAi screen to identify novel UPS regulators of Hh signaling. By assessing CiFL protein stabilization and *dpp-lacZ* reporter activity as simple but efficient readouts for Hh signaling, we identified two novel negative regulators of Hh signaling, each belonging to functionally distinct (E1 and E2) UPS complexes. Utilizing *in vivo* genetic and *in vitro* biochemical assays, we characterized these novel E1 and E2 genes as essential components of the neddylation pathway, which control the activity and stability of Cullin proteins and thereby regulate Ci protein stability and Hh signaling activity.

## Results

### The stability of endogenous Ci protein is regulated by the ubiquitin- proteasome system (UPS)

Ample genetic evidence highlights a role for UPS in the regulation of CiFL protein stability and activity, however, a previous report suggests that CiFL could also be degraded in the lysosome [31]. To distinguish the roles of these degradation pathways in the regulation of endogenous CiFL protein stability, we specifically prevented either UPS- or lysosome-mediated protein degradation, utilizing inhibitors in cultured fly cells as well as genetic manipulation in wing imaginal discs.

**Figure 1:**
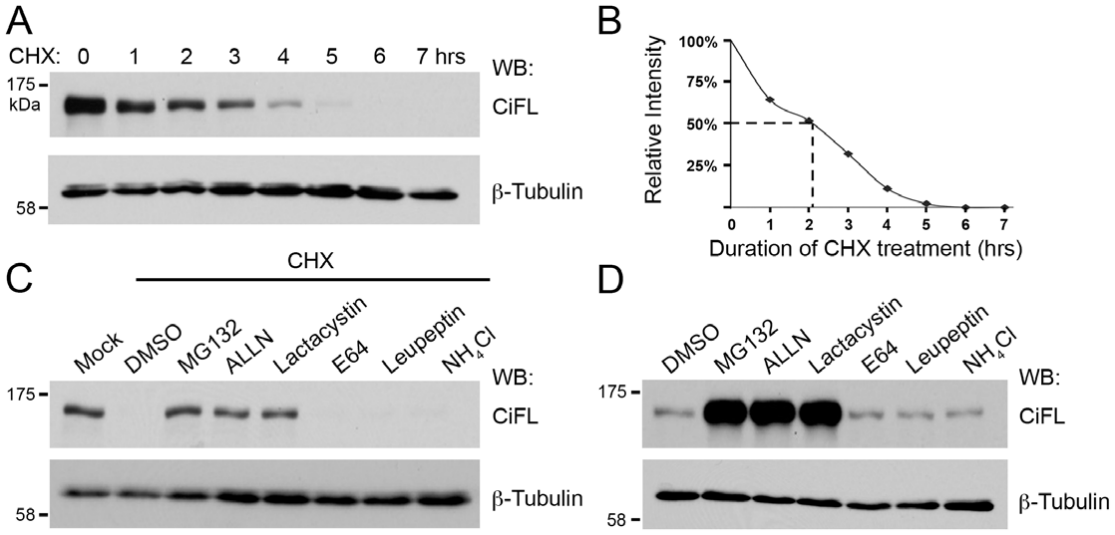
The stability of endogenous Ci is regulated by the UPS *in vitro*. (**A**) Lysates extracted from cl-8 cells that were treated with 50 µg/ml cycloheximide (CHX) for the indicated hours (hrs) were immunoblotted (WB) with the 2A1 antibody, which specifically recognizes full-length Ci (CiFL) [87]. β-Tubulin detection served as the loading control in all figures. All immunoblotting data presented in the figures are representative of independent experiments that were performed at least three times. (**B**) Endogenous CiFL degraded rapidly with a half-life of approximately two hours (indicated by dashed lines) as determined by Image J densitometry. (**C**) CHX-induced CiFL degradation was rescued upon pre-incubation with UPS inhibitors (MG132, ALLN and Lactacystin), but not with DMSO or lysosomal inhibitors (E64, Leupeptin and NH_4_Cl). (**D**) In the absence of CHX treatment, incubation with UPS inhibitors alone resulted in significant accumulation of CiFL, while lysosomal inhibitors had no effect, thus suggesting a physiological relevance of the UPS-mediated Ci degradation in Hh signaling.

First, we examined the half-life of endogenous CiFL protein in cl-8 cells, a fly cell line that is responsive to Hh signaling [27], [28], [32], [33]. When treated with cychloheximide (CHX), an inhibitor of nascent protein synthesis, CiFL protein exhibited a rapid turnover with a half-life of approximately two hours (Figure 1A and B). Next, we tested whether CiFL protein degradation is regulated by a UPS- or lysosome-mediated process. cl-8 cells were incubated with specific UPS inhibitors (MG132, ALLN or lactacystin) or lysosomal inhibitors (E64, leupeptin or NH_4_Cl) for three hours followed by CHX treatment for an additional six hours. We found that UPS inhibitors, but not lysosomal inhibitors, were able to protect CiFL protein from CHX treatment-induced degradation in cl-8 cells (Figure 1C). To demonstrate physiological relevance of UPS-mediated CiFL degradation, we examined the effect of these inhibitors in cl-8 cells without CHX treatment. Significant accumulation of CiFL was observed when the UPS activity was attenuated (Figure 1D). Taken together, our results suggest that the UPS played a major role in regulating CiFL stability *in vitro*.

**Figure 2:**
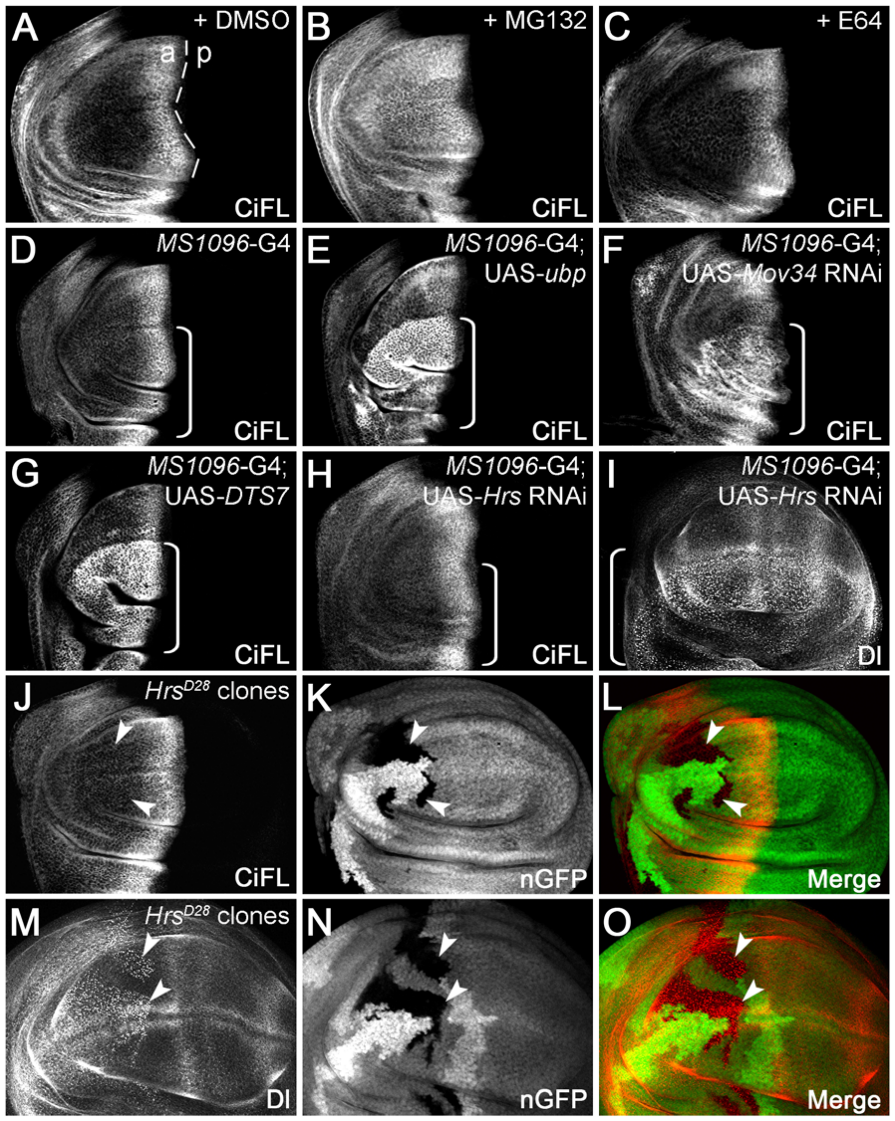
The stability of endogenous Ci is regulated by the UPS *in vivo*. (**A**–**C**) UPS inhibition protected CiFL from degradation in the wing disc. CiFL, detected with the 2A1 antibody, accumulated abutting the anterior/posterior (a/p) boundary (marked by the dashed line) of a wildtype wing disc treated with DMSO (**A**). Incubation with the UPS inhibitor MG132 led to accumulation of CiFL in more anterior cells in the wing disc (**B**), while the lysosomal inhibitor E64 had no obvious effect (**C**). (**D** and **E**) Blockage of ubiquitination in the wing disc by overexpressing UAS-*ubp* resulted in accumulation of CiFL in more anterior cells (**E**). *MS1096*-Gal4 (G4), which was used in Figures 2, 3, 4 and 6 to drive transgene expression at a much higher level in the dorsal compartment of the wing disc (indicated by a box bracket), did not alter the stability of CiFL (**D**). (**F**–**O**) Genetic manipulation to disrupt UPS- or lysosome-mediated protein degradation in wing discs. Knockdown of the 19S proteasome subunit *Mov34* by RNAi (**F**) or disrupting the function of the 20S proteasome core particle b2 subunit by expression of a dominant negative temperature-sensitive *DTS7* transgene (**G**) in the dorsal compartment of wing discs (indicated by box brackets) resulted in significant accumulation of CiFL. In contrast, the expression pattern of CiFL was not altered when the lysosomal function was disrupted by *Hrs* RNAi (**H**, box bracket) or in *Hrs^D28^* loss-of-function somatic clones (**J**–**L**, arrowheads). As a control, accumulation of Delta protein (Dl), which normally undergoes endocytosis to the lysosome, was observed when *Hrs* function was disrupted (**I**, **M**–**O**). *Hrs^D28^* loss-of-function clones were negatively marked by nuclear GFP (nGFP; **K** and **N**). Note that the *MS1096*-Gal4 driver alone did not alter the expression patterns of CiFL or Dl in wing discs (see Figure S1).

To investigate whether the stability of endogenous Ci is also subject to UPS control *in vivo*, we exposed *Drosophila* wing discs to inhibitors specific for either the UPS or the lysosome system. Hh protein is produced from cells present in the posterior compartment of the wing disc, and moves across the anterior/posterior (a/p) boundary to form a Hh morphogen gradient, thereby activating downstream target genes in anterior cells [1–4]. Those anterior cells abutting the a/p boundary receive the highest Hh signaling, while less signaling is transduced in the anterior-most cells. As a consequence, CiFL accumulates at a much higher level in anterior cells close to the a/p boundary (marked by the dashed line in Figure 2A), and levels sharply decline in more anterior cells. Consistent with our *in vitro* results, only the UPS inhibitors were sufficient to protect Ci protein from degradation in wing discs (Figure 2B and C; data not shown).

To further validate the results obtained from inhibitor studies, we specifically disrupted UPS or lysosome function in wing discs by genetic manipulation. First, we overexpressed a UAS-*ubp* transgene, which encodes a yeast DUB enzyme that has been used in several *Drosophila* studies to efficiently remove Ub from ubiquitinated substrates [34–36]. As expected, CiFL was stabilized in the dorsal compartment of the wing disc (indicated by a box bracket) when *ubp* transgene expression was driven by the *MS1096*-Gal4 driver (Figure 2E; cf. Figure 2D); *MS1096*-Gal4 driver confers transgene expression at a much higher level in the dorsal compartment of the wing disc (Figure S1C). This experiment suggests that CiFL is ubiquitinated under normal physiological conditions and degraded in the cells away from the Hh signaling source.

Since the UPS and lysosome can both recognize ubiquitinated proteins as substrates for degradation, we further distinguished these two pathways by directly inhibiting either the proteasomal or lysosomal pathway. The most common form of the proteasome is the 26S complex, which is composed of two major subcomplexes: the 19S regulatory particle and the 20S proteolytic core particle (reviewed in [15]). RNAi-mediated knockdown of two well-conserved 19S proteasome regulatory particle subunit genes, *Mov34* and *Rpn6* [37], resulted in significant accumulation of CiFL protein (Figure 2F; data not shown). Similarly, when the function of 20S proteasome core particle b6 (Pros26) or b2 (Prosbeta2) subunit was disrupted in the dorsal compartment of the wing disc by the expression of dominant negative temperature-sensitive mutants DTS5 or DTS7 [38], CiFL protein was stabilized in more anterior cells (Figure 2G; data not shown). In contrast, when the essential lysosomal components, *car* (*carnation*) [39], *dor* (dee*p orange*) [40], *Hrs* (*Hepatocyte growth factor regulated tyrosine kinase substrate*) or *Stam* (*Signal transducer adaptor molecule*) [41], [42], were specifically knocked down by RNAi, little if any effect on CiFL stability was observed (Figure 2H; data not shown). To rule out the possibility that the lack of Ci accumulation in the wing disc was due to the inefficiency of RNAi transgenes used, we generated loss-of-function somatic clones of lysosomal mutant alleles; clones were negatively marked by GFP (Figure 2K and N, arrowheads). Similar to our RNAi results, we found that the expression level of CiFL protein was un-altered in cells present in mutant clones (indicated by arrowheads) for *dor*, *Hrs* or *Stam* genes (Figure 2J–L; data not shown). As a control, Notch signaling ligand Delta (Dl) protein, which is normally endocytosed through lysosomal components [43], accumulated in the cells (i.e. dotted pattern, cf. Figure S1A) where lysosomal function was disrupted by *Hrs* RNAi or in loss-of-function *Hrs* mutant clones (Figure 2I and M–O). Taken together, the results from our *in vitro* and *in vivo* experiments strongly support a major role of UPS in regulating CiFL protein stability in *Drosophila*.

### Targeted *in vivo* RNAi screen to identify novel UPS genes in regulating Hh signaling

To study the function of UPS regulators in Hh signaling, we searched the fly genome and identified a set of proteins that contain UPS-related domains [44]. A single E1 (Uba1, CG1782) [45], [46] and a single E2 (UbcD6, CG2013) [47], [48] enzyme are believed to function in UPS-mediated protein degradation in *Drosophila*. Seven additional E1s and 33 E2s have been identified based on the presence of signature protein domains [44], [49], [50]. Among those, Uba2 (CG7528) and Aos1 (CG12276) are believed to form a dimer and activate the Small Ub-like Modifier (SUMO) protein in *Drosophila* [51], [52], while Ubc9 (CG3018) functions as the E2 conjugating enzyme in the fly SUMO pathway [52], [53]. The function of other E1s or E2s are largely unknown in *Drosophila*. In contrast to limited numbers of E1s and E2s, there is a large array of E3 ligases that are responsible for targeting specific substrates for degradation. Based on sequence homology of their E2-binding domains, E3s can be generally classified into two major subfamilies: HECT (the homologous to E6-AP carboxyl terminus) domain- and RING (the really interesting new gene) finger domain-containing E3s [17], [44]. We identified 14 HECT- and 134 RING-containing proteins in the fly genome. We also found a set of proteins containing other domains that normally contribute to the formation of the E3 complex, including the F-box domain, Cullin domain, N-recognin domain, SKP1 domain and U-box domain. In total, we identified 8 E1, 34 E2 and 207 E3 genes in the fly genome (Table 1).

**Table 1:** UPS genes identified in the fly genome.

Gene class	IPR domain	Number of genes	Number of RNAi lines
E1	IPR000594	8	12
E2	IPR000608	34	61
E3 (HECT)	IPR000569	14	25
E3 (RING)	IPR001841	134	219
E3 (Cullin)	IPR001373	7	14
E3 (F-Box)	IPR001810	37	59
E3 (SKP1)	IPR001232	8	13
E3 (U-Box)	IPR003613	5	9
E3 (N-recognin)	IPR003126	2	4
In total		248*	414*

*Note that CG15437 protein contains both an E2 domain and a F-Box domain.

The availability of two collections of transgenic RNAi libraries housed in the VDRC (Vienna *Drosophila* RNAi Center, Austria) [54] and the NIG-Fly Stock Center (National Institute of Genetics, Japan), makes it possible to screen nearly all UPS regulators that we identified for their effects on Hh signaling *in vivo*. We obtained 414 RNAi lines targeting 238 genes out of a total of 248 UPS genes (Table S1). Hh signaling functions as an important morphogen as well as a powerful mitogen during fly wing development. We therefore examined adult wing blade patterning and larval wing disc development as efficient and reliable readouts in our *in vivo* RNAi screen to identify Hh signaling-specific UPS genes.

We first knocked down the expression of individual UPS genes in wing discs by RNAi using the *MS1096*-Gal4 driver, and then examined the resulting adult wing blade phenotypes. We found that reducing the expression of 72 UPS genes altered adult wing morphology (Table S1). As several developmental signaling systems, including Hh, Wnt, TGF-β and Notch signaling, collaborate to control wing morphogenesis, a secondary screen was conducted to identify Hh signaling-specific UPS regulators. We evaluated the distribution patterns of CiFL in wing discs overexpressing UPS RNAi. To correlate Ci expression with Hh signaling activity, we also examined the expression of a *decapentaplegic* (*dpp*)*-lacZ* enhancer trap reporter; *dpp* is a direct transcriptional target of Hh signaling in the wing disc. To further validate our screen results, we investigated whether the effect of UPS regulation is direct by examining CiFL stability in UPS RNAi overexpressing clones (i.e. flip-out clones) [55], [56]. Only those UPS genes that cell-autonomously affect CiFL protein were chosen as true Ci regulators.

Our RNAi screen successfully identified E3 Ub ligase members which are known UPS regulators of CiFL stability [5–8], including *slimb*, *Cul1* (*lin 19*), and *Roc1a* (Figure S2; data not shown). These genes encode Slimb-Cul1 complex components that have been shown to destabilize CiFL by promoting proteasomal degradation. In addition, we identified two novel genes (*CG13343* and *CG7375*) that regulate CiFL stability and Hh signaling activity. To date, these genes are uncharacterized and their functions poorly understood. We therefore conducted genetic and biochemical studies to understand the molecular functions of these novel genes in *Drosophila*.

### CG13343 functions as a neddylation E1-activating enzyme in *Drosophila*

**Figure 3:**
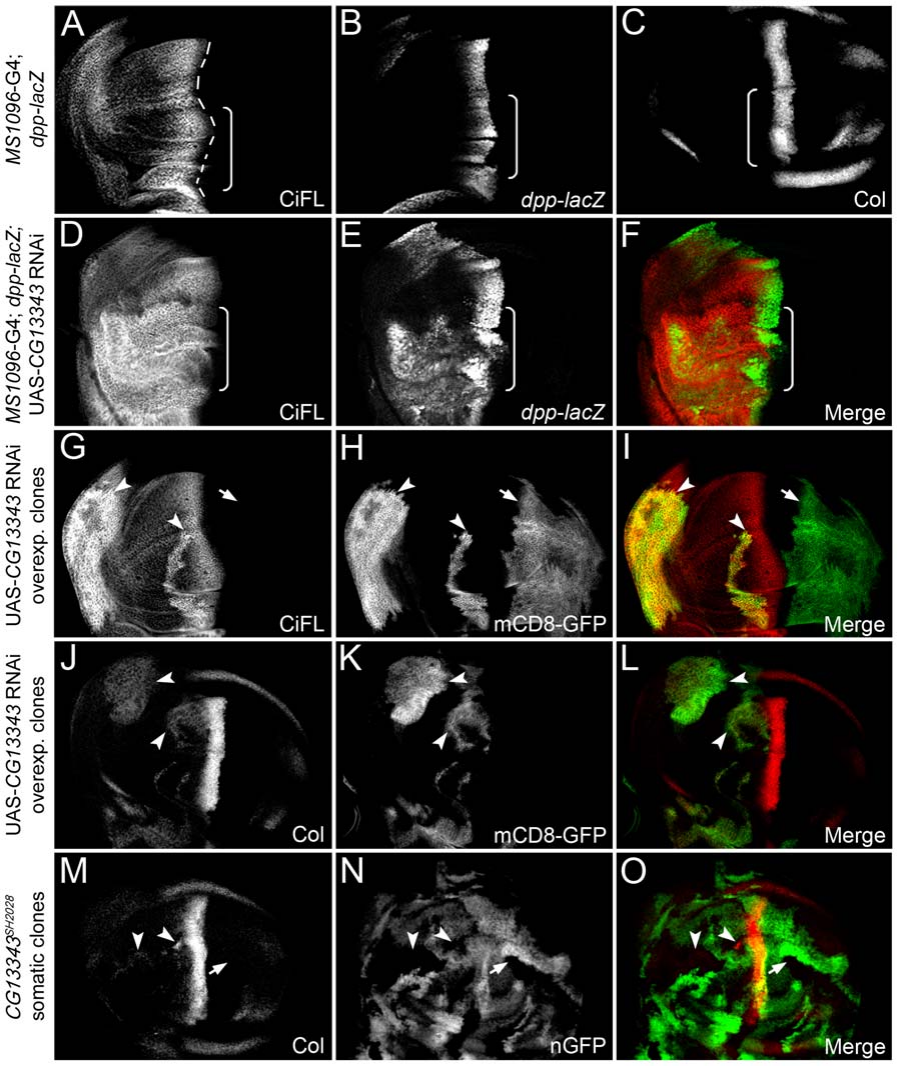
*CG13343* negatively regulates CiFL stability and Hh signaling. (**A**–**C**) Stabilization of CiFL (**A**), induction of Hh signaling reporter *dpp-lacZ* (**B**) and Col protein (**C**) abutting the a/p boundary (indicated by the dashed line in **A**) in wing discs correlate with low-, intermediate- and high-threshold Hh signaling activity, respectively. Box brackets mark the dorsal compartment of wing discs where *MS1096*-Gal4 exhibits a much higher activity (also see Figure S1C). (**D**–**O**) *CG13343* negatively regulates Hh signaling. RNAi knockdown of *CG13343* in the dorsal compartment of the wing disc (box bracket) led to accumulation of CiFL protein (**D**) and expansion of *dpp-lacZ* activity (**E**). Similarly, CiFL stabilization (**G**) and ectopic Col expression (**J**) were observed in *CG13343* RNAi-overexpressing cells (positively marked by mCD8-GFP in **H** and **K**) in anterior clones (**G**–**L**, arrowheads), but not in posterior clones (arrow). Ectopic Col activation was also evident in *CG13343^SH2028^* somatic clones (negatively marked by nGFP in **N**) located in the anterior compartment of the wing disc (**M**, arrowheads). Note that Ci and Col are not expressed in posterior cells.

The *CG13343* transcript was uniformly expressed in the wing disc (Figure S3B). Reduced expression of *CG13343* by RNAi in wing discs at 29°C resulted in lethality at the pupal stage. At 25°C, several escapers survived into adulthood, with their wing blades folded and damaged (data not shown). To accurately determine whether *CG13343* played a role in Hh signaling, we investigated the effect of *CG13343* knockdown in the wing disc, a primordium of the adult wing. During patterning in the developing wing, distinct target genes are activated in response to different strengths of Hh signaling activity (Figure 3A–C) [33]. Low-threshold Hh signaling stabilizes CiFL. The expression of one of these targets, *dpp*, is induced by intermediate-threshold Hh signaling, whereas two other targets, *ptc* and *collier* (*Col*), respond to high-threshold Hh signaling. Here, we examined the effects of reduced CG13343 activity on CiFL, *dpp-lacZ* reporter and Col protein in the wing disc.

Knockdown of *CG13343* expression by RNAi in the wing disc resulted in the stabilization of CiFL (Figure 3D) and the activation of Hh signaling: *dpp-lacZ* (Figure 3E) and Col protein (data not shown) were ectopically expressed. Notably, *CG13343* RNAi had little effect on the abundance of Ci75, the repressor form of Ci (Figure S4A). Further analyses of cells in *CG13343* RNAi overexpressing clones, which were positively marked by mCD8-GFP (Figure 3H and K), confirmed that *CG13343* functions cell autonomously to regulate CiFL stability (Figure 3G, arrowheads) and Col activation (Figure 3J, arrowheads). Three independent RNAi transgenic lines were tested and similar effects on CiFL stabilization and target gene activation were observed (Figure 3; data not shown). The efficiency and specificity of *CG13343* RNAi was examined by semi-quantitative RT-PCR (Figure S5A).

**Figure 4:**
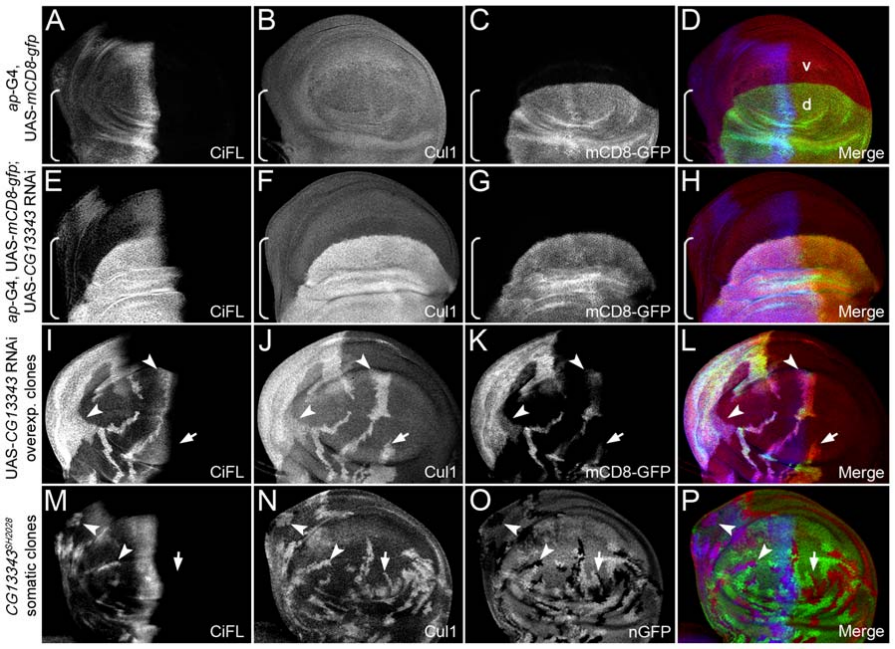
The stability of Cul1 is regulated by *CG13343*. (**A**–**D**) Uniform expression of Cul1 protein (**B**) in a wing disc expressing the *ap*-Gal4 driver. UAS-*mCD8-gfp* expression (**C**) reflects the *ap*-Gal4 activity in the dorsal (d) compartment (marked by the box bracket). (**E**–**P**) Regulation of Cul1 protein stability by *CG13343*. Knockdown of *CG13343* expression by RNAi in the dorsal compartment of the wind disc resulted in significant accumulation of CiFL (**E**) and Cul1 (**F**). Analysis of *CG13343* RNAi-overexpressing clones (positively marked by mCD8-GFP in **K**) in the anterior compartment (arrowheads) confirmed that the stability of CiFL (**I**) and Cul1 (**J**) was cell-autonomously regulated by *CG13343*. Similarly, stabilized CiFL (**M**, arrowheads) and Cul1 (**N**, arrowheads and arrow) were observed in *CG13343^SH2028^* loss-of-function somatic clones (negatively marked by nGFP in **O**) in the wing disc.

To validate the *CG13343* RNAi knockdown results, we generated loss-of-function somatic *CG13343^SH2028^* mutant clones in the wing disc, which were negatively marked by nGFP (Figure 3N). The *CG13343^SH2028^* is a recessive-lethal mutant that arose from a *P*-element insertion at the position 34 bps immediately after the ATG start codon in the *CG13343* locus [57]. RT-PCR results indicated that *CG13343^SH2028^* represented a null mutation for *CG13343* (Figure S5C). Consistent with the *CG13343* RNAi results, we found that CiFL was stabilized (Figure 4M) and Col was ectopically expressed in *CG13343^SH2028^* clones (Figure 3M, arrowheads), indicating that *CG13343* was required to control both low- and high-threshold Hh signaling.

Utilizing a protein domain search, we found that the CG13343 protein was highly conserved from yeast to human, and contain a Ub-activation domain normally found in Ub-activating E1 enzymes (Figure S6). Uba3 is the CG13343 ortholog in yeast and human, which functions as the NEDD8 activating E1 enzymes in the neddylation process [58]. The best-characterized substrates for neddylation are Cullin family proteins [18–22], [58]. Neddylation of Cullin proteins results in Cullin activation, but also leads to its own destabilization [18–22]. To investigate whether the function of CG13343 mimicked its yeast and vertebrate counterparts we examined the stability of Cullin proteins in wing discs. RNAi-mediated knockdown of *CG13343* was carried out in the dorsal compartment of wing discs using an *ap*-Gal4 driver (expression pattern of *ap*-Gal4 is shown in Figure 4C). Notably, only one commercially available antibody raised against vertebrate Cullins (i.e. α-Cul1) worked for immunohistochemistry in wing discs. As expected, Cul1 accumulated in dorsal compartment cells (marked by box brackets) where *CG13343* expression was down-regulated (Figure 4F, cf. Figure 4B). Consistent with this outcome, increased Cul1 protein expression (Figure 4J) was observed in clones overexpressing *CG13343* RNAi (i.e. mCD8-GFP-positive cells in Figure 4K) as well as in loss-of-function somatic *CG13343^SH2028^* clones (i.e. nGFP-negative cells in Figure 4N).

**Figure 5:**
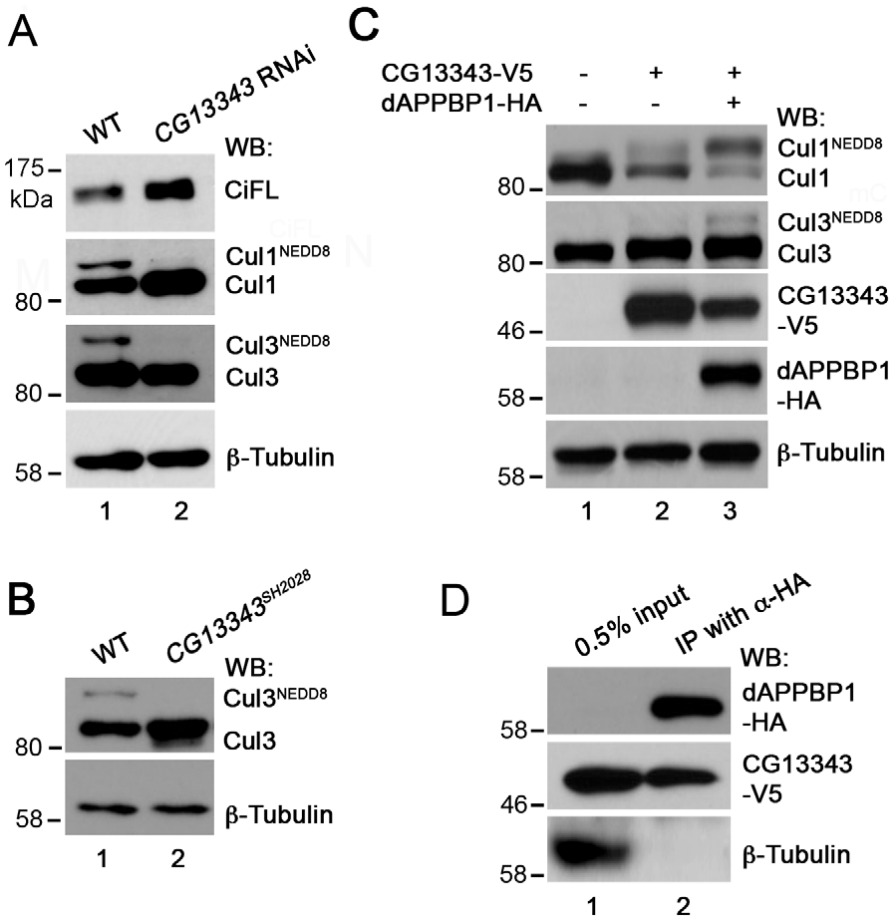
CG13343 protein functions as the E1 enzyme for Cullin neddylation. (**A**) Immunoblot analysis (WB) of lysates extracted from wildtype (WT) wing discs (lane 1) or wing discs overexpressing *CG13343* RNAi driven by the *MS1096*-Gal4 driver (lane 2). *CG13343* RNAi led to accumulation of both CiFL and Cul1 (lane 2). Furthermore, stabilized Cul1 was predominantly un-neddylated (lane 2). Neddylation of Cul3, another Cullin family protein, was also reduced. However, there was no accumulation of un-neddylated Cul3 (lane 2). Note that this Cul3 antibody does not work for immunohistochemistry in wing discs. (**B**) Immunoblot analysis of lysates extracted from wildtype (lane 1) or homozygous loss-of-function *CG13343^SH2028^* first-instar larvae (lane 2). Neddylation of Cul3 protein was abolished and un-neddylated Cul3 was stabilized. (**C**) The E1 activity of CG13343 for Cullin neddylation. In an *in vitro* neddylation assay, purified human Ubc12 was used as E2 and cl-8 cell lysate provided the source for Cullin proteins. In the absence of added *CG13343-V5*, minimal neddylation activity was observed (lane 1). Overexpressed CG13343-V5 in cl-8 cells was sufficient to function as an E1 enzyme to neddylate Cul1, but not Cul3 (lane 2). The neddylation activity of CG13343-V5 was greatly enhanced when *CG13343-V5* was co-expressed with *dAPPBP1-HA* in cl-8 cells (lane 3), resulting in the neddylation of both Cul1 and Cul3. Note that equal amounts of plasmid DNA were transfected in cl-8 cells, i.e. half amount of *CG13343-V5* plasmid was transfected in lane 3 compared to that in lane 2. (**D**) CG13343 protein forms an E1 complex with dAPPBP1. cl-8 cells were transiently transfected with *dAPPBP1-HA* and *CG13343-V5*, and 0.5% of the cell lysate was loaded as input (lane 1). An anti-HA antibody was used for immunoprecipitation (IP) (lane 2).

To investigate whether CG13343 protein was required for Cullin neddylation *in vivo*, we exploited the fact that neddylated Cullin migrates slower than its unmodified counterpart on SDS-PAGE [7], [22], [59]. To examine the extent of Cullin neddylation, antibodies specific for Cul1 and Cul3 were used. In wildtype wing disc lysates, both Cul1 and Cul3 proteins were neddylated (Figure 5A, lane 1). When *CG13343* expression in wing discs was knocked down by RNAi, which stabilized CiFL, we found that neddylation of Cul1 or Cul3 was largely reduced (lane 2). These results are consistent with previous reports illustrating that neddylated Cullins are required for Ci degradation [22], [59]. As neddylated and activated Cullins are less stable, we observed that wing disc lysates overexpressing *CG13343* RNAi had higher levels of Cul1, presumably resulting from stabilization of un-neddylated Cul1. However, it is interesting to note that the amount of un-neddylated Cul3 was not obviously changed in cells expressing *CG13343* RNAi. The different sensitivity between Cul1 and Cul3 stabilization in response to *CG13343* RNAi could be due to incomplete depletion of *CG13343*. To address this possibility, we examined the neddylation and stabilization of Cul3 in protein lysates extracted from first-instar larve homozygous of *CG13343^SH2028^*. As predicted, the accumulation of un-neddylated Cul3 protein was evident in the *CG13343^SH2028^* mutant (Figure 5B, lane 2). These results confirmed that CG13343 played a role in the neddylation pathway. To definitively demonstrate that CG13343 protein functioned as a neddylation E1 enzyme, we examined Cullin neddylation using a cell-free neddylation assay. Minimal neddylation on Cul1 or Cul3 was detected in fly cl-8 cell lysates after one-hour incubation in the presence of purified E2 enzyme, NEDD8 and ATP (Figure 5C, lane 1). This approach provided a relatively clean system for testing the capacity of CG13343 as a neddylation E1 enzyme *in vitro*. When V5-tagged CG13343 (CG13343-V5) was overexpressed in cl-8 cells, we observed obvious, albeit weak, neddylation on Cul1, but not on Cul3 (lane 2).

Structural studies reveal that human Uba3 forms a heterodimer with β-Amyloid precursor protein binding protein 1 (APPBP1), and together they function as an active neddylation E1 complex [18–21]. Genetic evidence suggests that the *Drosophila* homolog of APPBP1 (dAPPBP1) may participate in the neddylation process [59]. We found that, when co-expressed in cl-8 cells, CG13343 protein was able to form a complex with dAPPBP1 (Figure 5D). Furthermore, this complex sufficiently acted as a potent neddylation E1 enzyme to neddylate both Cul1 and Cul3 (Figure 5C, lane 3; cf. lane 2). Taken together, our genetic and biochemical assays provide strong evidence that CG13343 protein was a functional homolog of Uba3 as it acted together with dAPPBP1 to function as a NEDD8 E1-activating enzyme. Hence, we propose the renaming of CG13343 to dUba3 (*Drosophila* Uba3).

### CG7375 functions as a neddylation E2-conjugating enzyme in *Drosophila*

**Figure 6:**
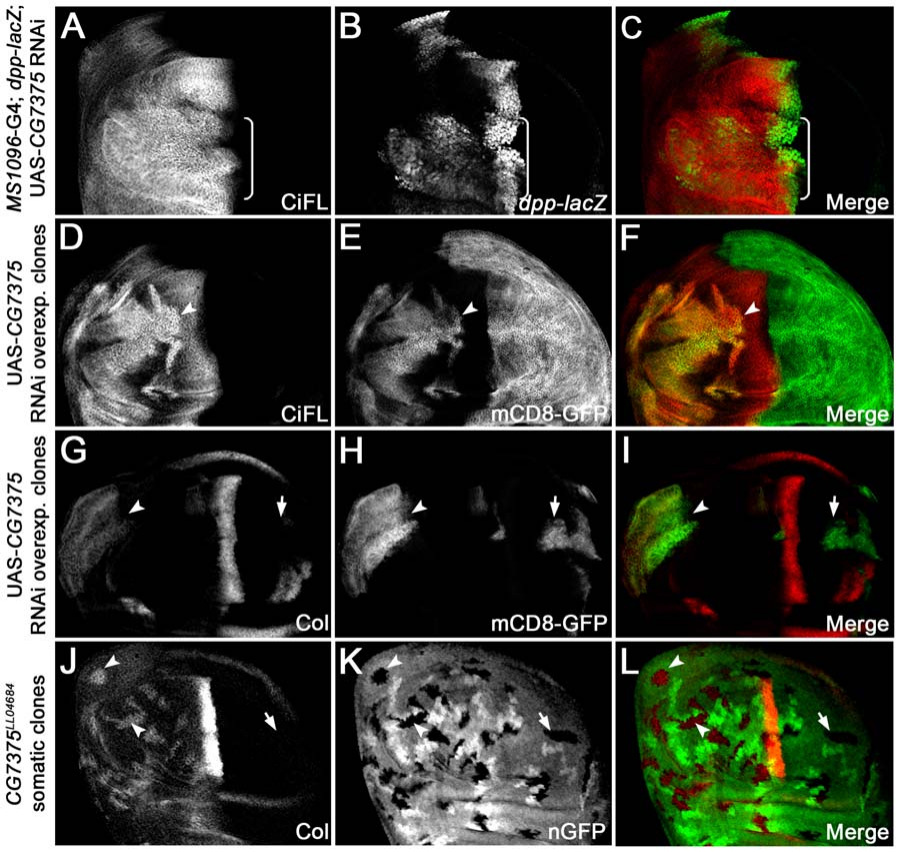
*CG7375* negatively regulates CiFL stability and Hh signaling. RNAi knockdown of *CG7375* in the dorsal compartment of the wing disc (box bracket) led to accumulation of CiFL (**A**) and expansion of *dpp-lacZ* (**B**). Analysis of *CG7375* RNAi-overexpressing clones (positively marked by mCD8-GFP in **E** and **H**) confirmed that CiFL stability (**D**, arrowhead) and Col expression (**G**, arrowhead) were cell-autonomously regulated by *CG7375* in the anterior compartment of the wing disc. Similarly, ectopic Col expression (**J**, arrowheads) was observed in loss-of-function *CG7375^LL04684^* somatic clones (negatively marked by nGFP in **K**) in the wing disc.

The second gene identified from our targeted RNAi screen was *CG7375*. Similar to *CG13343*, *CG7375* is uniformly expressed in the wing disc (Figure S3E). knockdown of *CG7375* expression by RNAi in the dorsal compartment of wing discs activated Hh signaling: elevated CiFL protein stabilization (Figure 6A and Figure S4B) as well as expanded *dpp-lacZ* (Figure 6B) and Col expression (data not shown) were observed. Analysis of cells in *CG7375* RNAi overexpressing clones revealed that *CG7375* acted cell autonomously to regulate CiFL stability (Figure 6D, arrowhead) and Col expression (Figure 6G, arrowhead).

**Figure 7:**
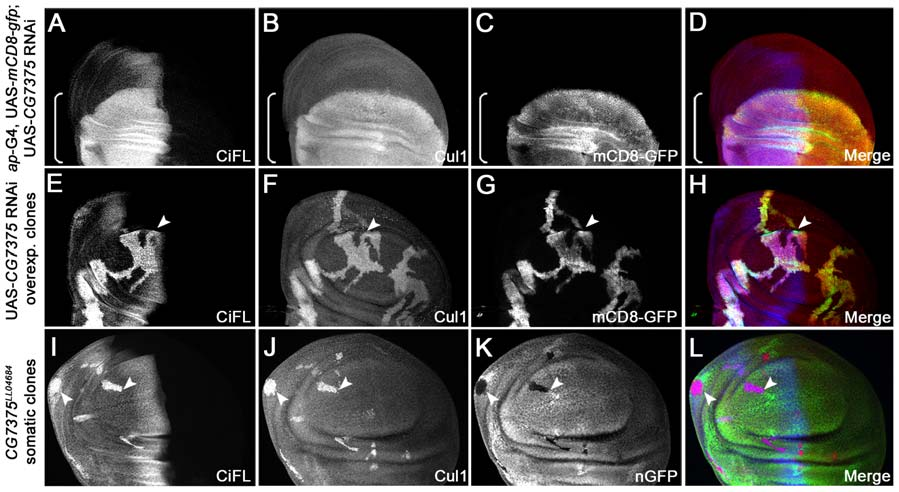
*CG7375* controls Cul1 protein stability. (**A**–**D**) Knockdown of *CG7375* expression by RNAi in the dorsal compartment of the wing disc (marked by mCD8-GFP expression in **C**) resulted in significant accumulation of CiFL (**A**) and Cul1 (**B**). (**E**–**L**) Cell-autonomous stabilization of CiFL (**E** and **I**) and Cul1 (**F** and **J**) was observed when *CG7375* function was disrupted in *CG7375* RNAi-overexpressing clones (arrowhead in **E**–**H**; positively marked by mCD8-GFP in **G**) or in *CG7375^LL04684^* loss-of-function somatic clones in wing discs (arrowheads in **I**–**L**; negatively marked by nGFP in **K**).

To validate the *CG7375* RNAi knockdown results, we generated loss-of-function somatic *CG7375^LL04684^* mutant clones in the wing disc, which were negatively marked by nGFP (Figure 6K). The *CG7375^LL04684^* is a recessive-lethal mutant that arose from a *piggyBac* insertion at the position 19 bps immediately after the ATG start codon in the *CG7375* locus [60]. RT-PCR results indicated that *CG7375^LL04684^* represented a null mutation for *CG7375* (Figure S5D). Consistent with the *CG7375* RNAi results, we found that CiFL was stabilized (Figure 7I) and Col was activated in *CG7375^LL04684^* clones (Figure 6J, arrowheads). These results suggested that *CG7375* behaved similarly to *CG13343* to regulate Ci stability and Hh signaling activation.

CG7375 protein harbors two distinct functional motifs: the E1 binding motif and E2 activity core (Figure S7). Recently, CG7375 has been predicted to be involved in neddylation, most likely acting as an E2 NEDD8 conjugating enzyme [61]. This hypothesis partially relies on the fact that CG7375 contains a small N-terminal extension shared only by E2 Ubc12 family members specific for NEDD8 conjugation [62]. However, to date, no functional studies have been conducted to demonstrate that CG7375 acts as the *Drosophila* NEDD8 E2 enzymes [61], [63].

To determine whether CG7375 protein functions in fly neddylation, we first examined the expression pattern of Cul1 in wing discs where *CG7375* expression was reduced. Both CiFL and Cul1 protein levels were significantly increased in the dorsal compartment of the disc where *CG7375* RNAi was overexpressed by the *ap*-Gal4 driver (Figure 7A–D). Utilizing *CG7375* RNAi overexpressing clones (Figure 7E–H, arrowhead) as well as loss-of-function somatic *CG7375^LL04684^* clones (Figure 7I–L, arrowheads), we demonstrated that the effect of CG7375 on the stabilization of Ci and Cul1 was cell autonomous.

**Figure 8:**
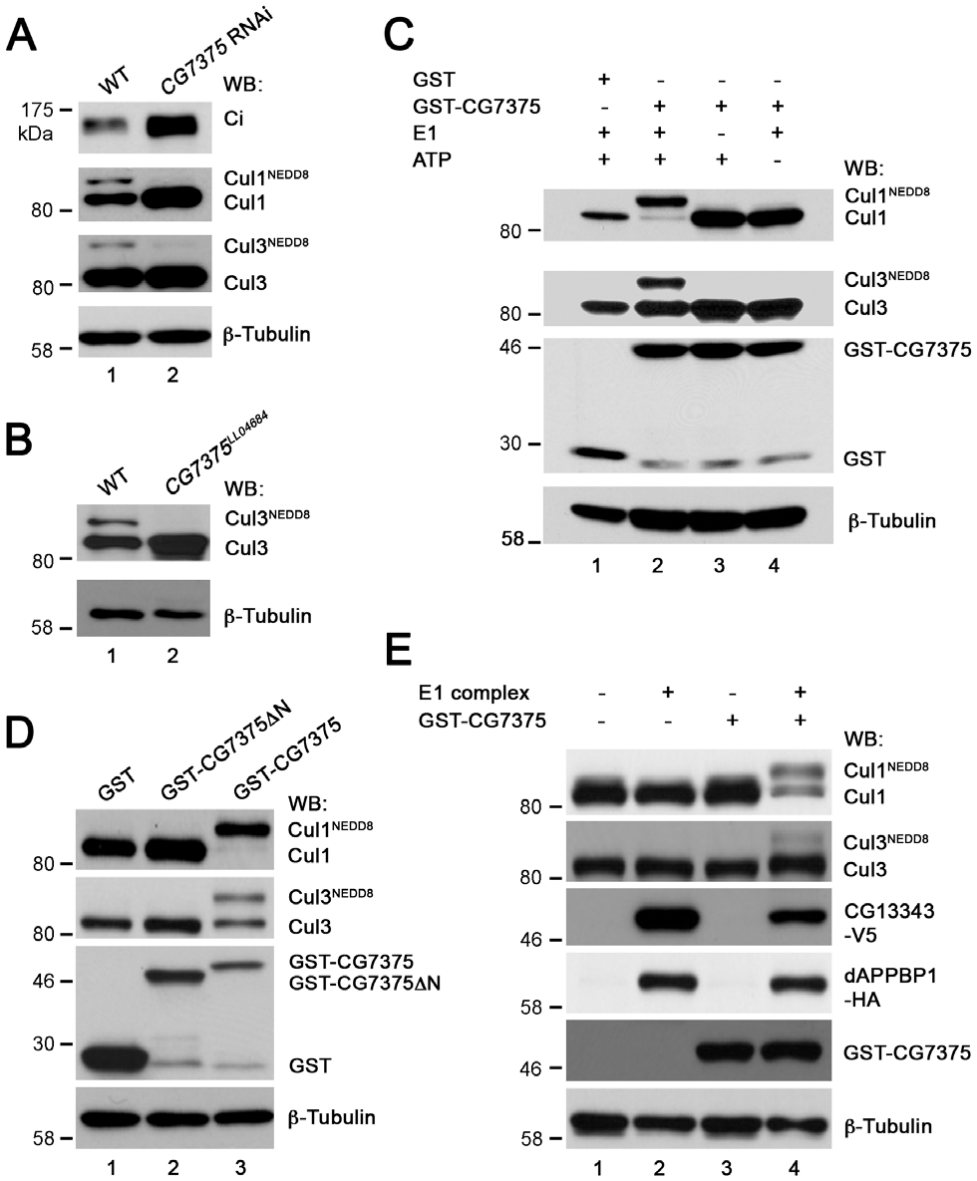
CG7375 protein functions as the E2 enzyme for Cullin neddylation. (**A**) Immunoblot analysis (WB) of lysates extracted from wildtype (WT) wing discs (lane 1) or wing discs overexpressing *CG7375* RNAi driven by the *MS1096*-Gal4 driver (lane 2). *CG7375* RNAi led to accumulation of both CiFL and un-neddylated Cul1 (lane 2). Similarly, neddylation of Cul3 was reduced, but the amount of un-neddylated Cul3 was not obviously changed (lane 2). (**B**) Immunoblot analysis of lysates extracted from wildtype (lane 1) or homozygous *CG7375^LL04684^* first-instar larvae (lane 2). Neddylation of Cul3 protein was abolished and un-neddylated Cul3 was stabilized. (**C** and **D**) The E2 activity of CG7375 for Cullin neddylation. In an *in vitro* neddylation assay, purified human Uba3/APPBP1 complex was used as E1 and lysates extracted from wing discs expressing *CG7375* RNAi provided the source for Cullin proteins. *CG7375* RNAi wing disc lysates did not display neddylation activity (as 90% endogenous *CG7375* was knocked down by *CG7375* RNAi; Figure S5C), unless purified GST-CG7375 protein was added (lane 2 in **C**; lane 3 in **D**): both Cu1 and Cul3 were neddylated. Purified GST protein was used as a negative control (lane 1 in **C** and **D**). The neddylation activity of GST-CG7375 was dependent on the presence of purified E1 complex (lane 3 in **C**) and ATP (lane 4 in **C**). The N-terminus of human ortholog of CG7375 (Ubc12) is required to selectively recruit NEDD8's E1 to promote thioester formation between E2 and NEDD8 (Figure S7). Deletion of this conserved N terminal motif in GST-CG7375DN abolished its neddylation E2 activity (lane 2 in **D**). (**E**) *In vitro* reconstitution of *Drosophila* neddylation cascade. Cul1 and Cul3 were neddylated when both E1 complex (CG13343-V5 and dAPPBP1-HA produced in cl-8 cells) and E2 enzyme (GST-CG7375) were added to cl-8 lysates, which provided the source of Cullins (lane 4). Adding E1 (lane 2) or E2 (lane 3) alone did not result in neddylation of Cul1 or Cul3.

To examine whether the elevated Cullin expression in wing discs was due to reduced neddylation, we compared Cullin neddylation in protein lysates extracted from wing discs with or without overexpressed *CG7375* RNAi. As expected, Cul1 neddylation was significantly reduced (Figure 8A, lane 2; cf. lane 1), which resulted in significant accumulation of Cul1 in wing discs expressing *CG7375* RNAi (Figure 7). However, *CG7375* RNAi had little effect on the stabilization of un-neddylated Cul3 (lane 2). Consistent with the result observed in *dUba3* loss-of-function larvae (Figure 5B), we found that un-neddylated Cul3 accumulated in protein lysates extracted from the loss-of-function *CG7375^LL04684^* mutant larvae (Figure 8B, lane 2).

To demonstrate that CG7375 protein functions as a neddylation E2 enzyme, we tested whether CG7375 protein could transfer NEDD8 to Cullin proteins in an *in vitro* cell-free assay. Cullins were provided from lysates extracted from wing discs whose endogenous *CG7375* mRNA was significantly reduced by *CG7375* RNAi (Figure S5B). In this situation, neither Cul1 nor Cul3 was notably neddylated (Figure 8A, lane 2). Addition of GST protein alone to the lysates did not result in Cullin neddylation (Figure 8C, lane 1). In contrast, addition of purified GST-CG7375 fusion protein was sufficient to conjugate NEDD8 to almost all Cul1 protein present in wing disc lysates (lane 2). Furthermore, neddylation was dependent on the presence of a functional E1 enzyme complex (lane 3) and ATP (lane 4). The ability of CG7375 to conjugate NEDD8 was also true for Cul3, although the overall degree of Cul3 neddylation was weaker than that of Cul1 (lane 2).

Recent crystal structure studies on the human E2 neddylation enzyme, Ubc12, suggest that a unique motif present in the N terminus of the proteins (Figure S7) is crucial for recruiting NEDD8's E1 enzyme to promote thioester formation between Ubc12 and NEDD8 [62]. We therefore tested whether this motif plays a conserved role in *Drosophila*. We found that deletion of the characteristic N terminal motif (CG7375DN) completely abolished CG7375 protein's neddylation E2 activity (Figure 8D, lane 2). Together, our genetic and *in vitro* biochemical analyses demonstrate that CG7375 is a *bona fide* NEDD8 E2-conjugating enzyme. Thus, we propose the renaming of CG7375 to dUbc12.

In vertebrates, both Uba3-APPBP1 and Ubc12 are required for NEDD8 conjugation to Cullin proteins. Consistent with this, CG13343-dAPPBP1 E1 complex (Figure 8E, lane 2) or purified GST-CG7375 alone (lane 3) was unable to promote Cul1 or Cul3 neddylation in a cell-free neddylation assay, unless both enzymes were present (lane 4). These experiments indicate that dUba3 (i.e. CG13343) and dUbc12 (i.e. CG7375) function together in an enzyme cascade for neddylation in *Drosophila*.

## Discussion

In this study, we utilized a targeted RNAi screen and identified several candidate UPS regulators in patterning of the *Drosophila* wing. Focused investigation on two candidate genes, *CG13343* and *CG7375*, demonstrated that they played a critical role in Hh signal transduction by controlling the stability of Hh signaling transcription factor Ci to regulate both low- and high-threshold Hh signaling. Importantly, we provided genetic and biochemical evidence that protein products of these two genes participated in a conserved protein degradation process in *Drosophila*, functioning as the NEDD8 E1-activating and E2-conjugating enzymes in neddylation, respectively. Consistent with our biochemical analysis, reduction of dNEDD8 modifier was able to elicit a full spectrum of Hh pathway responses in the wing disc (Figure S8). Thus, we propose a model whereby the neddylation pathway negatively regulates Hh signaling at the level of Ci stability (Fig. S9). The activity of Cul1-based E3 ubiquitin ligase complex is activated by neddylation, which in turn promotes proteolytic cleavage of CiFL to Ci75, thereby antagonizing low-to-intermediate threshold Hh signaling. On the other hand, neddylation activates Cul3-based E3 ubiquitin ligase complex, which degrades CiFL to prevent high-threshold Hh signaling.

### A general requirement of the UPS in the regulation of Ci protein stability

Hh signaling activates downstream target genes in a de-repression manner, thereby protecting the transcription factor Ci from degradation and/or processing. Two distinct ubiquitin ligase complexes, Slimb-Cul1 and Rdx-Cul3, have been identified as key regulators of Ci stability [5–13]. Both complexes recognize CiFL as the substrate, targeting it for either partial or complete degradation. Two subcellular compartments, the lysosome and proteasome, are important for regulated protein degradation. Although there is general consensus that Ci degradation takes place in the proteasome, *in vivo* evidence directly demonstrating the requirement of the proteasome for endogenous Ci degradation is lacking.

Here, we examined the stability of endogenous CiFL when the UPS or lysosome function was disrupted either by treating cultured fly cells with specific inhibitors or by genetically manipulating wing discs. Our data, consistent with previous studies on Ci degradation/processing [5–13], strongly support a major role of the UPS in controlling endogenous CiFL stability. However, this conclusion is in direct conflict with a previous study by Dai et al., suggesting that a multivesicular body-localizing protein Debra (Dbr) might direct CiFL degradation to the lysosome [31].

To solve this apparent discrepancy, we carefully compare the experimental conditions we employed to examine CiFL stability in cl-8 cells with those in Dai *et al.* Ectopically expressed HA-CiFL in Dai *et al.* exhibits a half-life of 15 hours, which is significantly longer than that of endogenous CiFL (approximately two hours, Figure 1B) in this study as well as that of the overexpressed Myc-CiFL (approximately three hours) demonstrated by Jia et al. [64]. Thus, it is not surprising that ectopic HA-CiFL was unable to respond to either UPS inhibitor MG132 or lysosomal inhibitor E64 treatment in cl-8 cells unless additional Dbr proteins were provided. In contrast, endogenous CiFL in cl-8 cells (Figure 1) and in wing discs (Figure 2), as well as over-expressed Myc-CiFL in cl-8 cells [7], [10], [64] can be readily protected from degradation by inhibiting the UPS function. Our conclusion is further supported by the fact that, overexpressed Gli1, one of the vertebrate orthologs of Ci, is also subject to proteasomal regulation [65]. We suspect that the Dbr-mediated lysosomal degradation of HA-CiFL may reflect a backup/alternative mechanism when UPS regulation is overwhelmed by highly overexpressed HA-CiFL *in vitro*. In the future, it will be interesting to investigate the molecular mechanism of Dbr-mediated degradation of endogenous Ci, and more importantly, the relationship with the UPS-regulated Ci degradation *in vivo*.

### Differential neddylation of Cul1 and Cul3 in Hh signaling

Our work, together with other studies [22], [59], [66], [67], demonstrates that the activities of both Cul1 and Cul3 are controlled by neddylation in *Drosophila*. However, it should be noted that there are differences in their respective neddylation patterns in response to reduced neddylation. In hypomorphic *dUba3* or *dUbc12* RNAi-expressing wing discs, reduced neddylation led to high-level accumulation of un-neddylated Cul1, consistent with the notion that neddylated Cullin proteins are unstable [20]. However, the levels of un-neddylated Cul3 in this sensitized background seemed to be unaffected in wing disc lysates, although the reduction of neddylated Cul3 was evident (Figure 5A and Figure 8A). In contrast, when the neddylation process was compromised in *dUba3* or *dUbc12* mutant larvae, both Cul proteins were stabilized (Figure 5B and Figure 8B; data not shown). Similarly, much less neddylation of Cul3 is observed than that for Cul1 in our *in vitro* neddylation assays (Figure 5C and Figure 8C–E) as well as in vertebrate Cullins when tested in an *in vitro* assay [68]. This differential regulation of Cul1 and Cul3 is also observed in the fly mutants of *CSN5* and *Int6*, two genes that are essential for de-neddylation [22], [66]. Although our results could simply reflect that Cul3 neddylation requires a much higher neddylation activity than Cul1, we believe that intrinsic differences may exist between Cul1 and Cul3 proteins. Neddylated Cul3 might degrade more rapidly than neddylated Cul1, which could explain distinct behaviors of Cul1 and Cul3 in our study. Indeed, the percentage of neddylated Cul3 in the total pool of Cul3 proteins in wildtype wing discs (i.e. 50% lower as determined by Image J densitometry in this study) and in brain lobes and eye discs [67] is significantly lower than that of Cul1, suggesting differential stability of neddylated Cullins. Further analyses are required to test this hypothesis and to elucidate the functional significance of differentially regulated Cullin proteins.

Cul1 and Cul3 are required for regulating Ci stability, but they function in very different manners (Figure S9). The Slimb-Cul1 complex destabilizes Ci in the absence of Hh signaling through direct binding between Slimb and phosphorylated Ci [6], [7]. Hh signaling prevents Ci phosphorylation and thus protects Ci from Slimb-Cul1 mediated degradation, as seen in the cells in the anterior compartment of wing and eye discs that receive Hh from the posterior compartment. The Rdx-Cul3 complex, on the other hand, constitutively degrades Ci independent of phosphorylation modifications even in the presence of Hh signaling [9], [10], [69]–[71]. Therefore, the activity of the Rdx-Cul3 complex has to be strictly controlled to ensure a proper Hh signaling outcome. One way to restrict Rdx-Cul3 activity is to utilize Rdx as a direct Hh signaling target [9], [10], [70]. In cells receiving low to intermediate levels of Hh signaling, Rdx is not expressed. In cells receiving the highest level of Hh signaling, Rdx expression is induced, thus allowing the formation of Rdx-Cul3 complex to degrade un-phosphorylated Ci. As the result, cells abutting the a/p boundary in the wing disc and posterior to the morphogenic furrow in the eye disc express much lower levels of Ci. Maintaining a low but steady level of Ci in these cells is crucial for transducing high-threshold Hh signaling, as further downregulation or abnormal accumulation of Ci proteins leads to patterning defects in the wing and eye [9], [10], [69], [70]. Our hypothesis that neddylated Cul3 is highly labile may, in part, provide a solution. We believe that neddylated Cul3 could act as an intrinsic brake to prevent Ci from complete degradation by the Rdx-Cul3 complex. Interestingly, a similar mechanism may also exist in the regulation of the cyclin E activity. Phosphorylated cyclin E is subject to Cul1-mediated degradation [72–75], whilst a Cul3-based complex targets cyclin E for ubiquitination independent of protein phosphorylation [76], [77]. Further studies will reveal the impact of such differential activity of neddylated Cul1 and Cul3 in Hh signaling as well as cell cycle control.

### A conserved role of the neddylation process in regulating developmental signaling

NEDD8 was originally identified as one of a set of genes that is highly expressed in the embryonic mouse brain and was found to be down-regulated during development [78]. Subsequently, it was realized that NEDD8 is a Ub-like (UBL) protein, and is highly conserved in eukaryotes (reviewed in [79]). NEDD8 is ubiquitously expressed in most tissues and is essential for the viability of most model organisms (reviewed in [80]). Among the UBL family proteins, NEDD8 exhibits the highest protein sequence similarity with Ub and is conjugated to substrate proteins through a very similar enzyme cascade. However, the neddylation process utilizes its own set of enzymes to insure a specific conjugation pathway. Contrary to Ub, which is processed by a single E1 protein Uba1, a heterodimer of APPBP1 and Uba3 is required for NEDD8 activation. APPBP1 is homologous to the N-terminus of the Uba1 protein, whereas Uba3 is to the C-terminus [18–20], [49]. Studies in several organisms indicate that Ubc12 functions exclusively as the NEDD8 E2 enzyme [50], [78]–[80]. Much is known about the importance of the neddylation pathway in the regulation of developmental processes in *Drosophila* [6], [22], [59], [63], [66], [67], [69], but neither the identities nor the mechanisms of the fly NEDD8 E1 and E2 enzymes are known.

Our genetic and biochemical analyses demonstrate that CG13343 and CG7375 are functional orthologs of Uba3 and Ubc12 in *Drosophila*. Ubiquitous knockdown of either *dUba3* or *dUbc12* by driving RNAi transgenes with *tub*-Gal4 or *act*-Gal4 results in early larval lethality (data not shown). Similarly, homozygous mutants of the *CG13343^SH2028^ or CG7375^LL04684^* allele die at early larval stages (data not shown). Our results are consistent with observations that null mutants of *dNEDD8*, *dAPPBP1*, or components of the de-neddylation CSN complex also die in early larval stages [6], [13], [22], [59], highlighting a critical role of neddylation in normal animal development.

The best-characterized neddylation substrates are the Cullin family proteins, which serve as the scaffold for the SCF ubiquitin E3 complexes. The SCF E3s regulate numerous developmentally important substrates, such as cell cycle regulator cyclin E [72–77] and signaling transduction effectors, including Ci [5–13] and Armadillo/β-catenin [81], [82]. NEDD8 has also been implicated in transcriptional regulation, by neddylating another substrate, the p53 tumor suppressor protein; neddylated p53 inhibits its own transcription activity [83], [84]. The number of identified NE88-target proteins is growing and interestingly a recent study in *Drosophila* indicates that many non-Cullin proteins can be neddylated *in vivo* [85]. The mechanisms regulating the neddylation pathway and the roles these processes in modulating animal development is more complicated than we previously anticipated. Further studies of *dUba3* and *dUbc12* in a highly amenable genetic model system, like *Drosophila*, will contribute substantially to our understanding of how neddylation functions in development.

## Materials and Methods

### Fly genetics

*Act5C*>*yw*>Gal4, *ap*-Gal4, *MS1096*-Gal4, and *dpp-lacZ* were described previously [33], [55]. Transgenic RNAi flies targeting predicted UPS genes (Table S1) were obtained from the Vienna *Drosophila* RNAi Center (VDRC) [54] and the Fly Stocks of National Institute of Genetics (NIG-Fly). Targeted RNAi screen was conducted by crossing individual RNAi lines with *MS1096*-Gal4 at 29°C for altered adult wing blade morphogenesis. For those lines displaying defective wing patterning, CiFL protein stabilization and *dpp-lacZ* induction were examined in third-instar larval wing discs for their effects on Hh signaling.

Specific fly strains and cross conditions as shown in the figures are listed below. For Figure 2E and F, UAS-*ubp* (gift of Liqun Luo) [34], UAS-*Mov34* RNAi (V26183) or UAS-*Rpn6* RNAi (V18021) was crossed with *MS1096*-Gal4 at 18°C. For Figure 2G, UAS-*DTS5* or UAS-*DTS7* [38] was crossed with *MS1096*-Gal4 at 29°C. For Figure 2H and I, UAS-*Hrs* RNAi (Bloomington 28964 and 28026), UAS-*dor* RNAi (V33733 and V107053), UAS-*car* RNAi (TRiP HMS00972) or UAS-STAM RNAi (V22497) was crossed with *MS1096*-Gal4 at 29°C. For Figure 2J–O, second-instar larvae from the crosses *hs-flp;; ubi*-*gfp*, FRT40A×*Hrs^D28^*, FRT40A/Gla, Bc (gift of Hugo Bellen) [41], or *hs-flp;; ubi*-*gfp*, FRT40A×*STAM^2L3297^*, FRT40A/CyO (gift of Markus Affolter) [42], or *ubi*-*gfp*, *hs-flp*, FRT19A×*dor^8^*, FRT19A/FM7 (gift of Helmut Krämer) [39] were heat-shocked at 37°C for one hour to generate loss-of-function somatic clones in the wing disc. For Figure 3D–F, UAS-*CG13343* RNAi lines (V17137, V17139 and V105141) were crossed with *MS1096*-Gal4; *dpp-lacZ*/CyO at 29°C. For Figure 3G–L and Figure 4 I–L, overexpressing (“flip-out”) clones were generated by heat-shocking second-instar larvae from the crosses of *hs-flp*; *Act5C>yw*>Gal4, UAS-*mCD8-gfp*×UAS-*CG13343* RNAi lines at 37°C for one hour. For Figure 3M–O and 4M–P, late second-instar progeny of the cross *hs-flp*; FRT42D, *ubi-gfp*×FRT42D, *CG13343^SH2028^*/CyO (*Drosophila* Genetic Resource Center at Kyoto, 122114) were heat-shocked at 37°C for one hour. For Figure 4E–H, UAS-*CG13343* RNAi lines were crossed with *ap*-Gal4, UAS-*mCD8-gfp* at 29°C. For Figure 6D–I and Figure 7E–H, the same heat-shocking condition was used as for Figure 4I–L. For Figure 6A–C, UAS-*CG7375* RNAi (V35219, V35220 and V100761) was crossed with *MS1096*-Gal4; *dpp-lacZ*/CyO at 29°C. For Figure 7A–D, UAS-*CG7375* RNAi lines were crossed with *ap*-Gal4, UAS-*mCD8-gfp* at 29°C. For Figure 6J–L and 7I–L, late second-instar progeny of the cross *hs-flp;; ubi*-*gfp*, FRT2A×*CG7375^LL04684^*, FRT2A / TM6B (*Drosophila* Genetic Resource Center at Kyoto, 141316) were heat-shocked at 37°C for one hour. For Figure S8, late second-instar progeny of the cross *hs-flp*; *ubi-gfp*, FRT40A×*dNEDD8^AN015^*, FRT40A / CyO (gift of Cheng-Ting Chien) [6] were heat-shocked at 37°C for one hour.

### Molecular biology

Standard PCR method was used to amplify *CG13343*, *CG7375* and *dAPPBP1* coding sequences using cDNAs synthesized with mRNAs extracted from *yw* third-instar larvae. *CG13343-V5* and *dAPPBP1-HA* were cloned into pIZ-V5 vector (Invitrogen) for overexpressing in cl-8 cells. *CG7375* or *CG7375ΔN* (amino acids 2–23 were deleted) were cloned into pGST- parallel2 vector for generating GST-fusion proteins. Primers used are listed as follows: 5′-GTACGAATTCATGTCTGTCCACTCACCC-3′ and 5′-CTGATCTAGATAGACCATCTCCACCTCATT-3′ for *CG13343*; 5′-ATGCGAATTCTATGTCCTCGCCAGCCCCC-3′ and 5′-TCAGTCTAGATTAGAGGCTAGCGTAATCAGGAACGTCGTAAGGGTATAGCTTCAATGTGACACT-3′ for *dAPPBP1*; 5′- AGTCGAATTCAAATGATTAAACTATTCACG-3′ and 5′-CATGCTCGAGTCACTTGAGCAGACAGCACTC-3′ for *CG7375*; 5′-AGTCGAATTCAAATGGCGTCCGCCGCCCAGCTG-3′ and 5′- CATGCTCGAGTCACTTGAGCAGACAGCACTC-3′ for *CG7375ΔN*.

RT-PCR was used to measure the abundance of *CG13343* and *CG7375* mRNA after RNAi manipulation or in loss-of-function mutant alleles. To test RNAi efficiency, RNA were extracted from wing discs (100 pairs per sample) of third-instar larvae that expressed RNAi transgene under the control of the *MS1096*-Gal4 driver at 29°C. For characterization of loss-of-function alleles, RNA were extracted from GFP-negative first-instar larvae (40 larvae per sample) of *CG13343^SH2028^*/CyO, *Kr*-*gfp* or *CG7375^LL04684^*/TM3, *twi-gfp* flies. Total RNA was isolated with TRIzol reagent (Invitrogen) according to the manufacturer's protocol. Contaminated DNA was digested using RNase-free DNase followed by a phenol/chloroform extraction to remove protein. First strand cDNA was synthesized from 1 µg of each sample using SuperScript III reverse transcriptase (Invitrogen). Semi-quantitative PCR was performed utilizing 20–35 cycles. The linear amplification stage for each primer set was determined by running the same volumes of amplified products on an agarose gel. *α-tubulin* primers were used for loading control. Primers used are listed as follows: 5′-GGCGTTGTCAAGCACATCATTC-3′ and 5′-TTTATCACATCCTCCAGCGTGG-3′ for *CG13343* RNAi; 5′-GTACGAATTCATGTCTGTCCACTCACCC-3′ and 5′-CTGATCTAGATAGACCATCTCCACCTCAT-3′ to amplify full-length *CG13343* cDNA in *CG13343^SH2028^* mutant; 5′-GGAARCCAGTGCTGAACATCAACTC-3′ and 5′-ACGCATCGCCTTCTTTACATTG-3′ for CG7375 RNAi; 5′-AGTCGAATTCAAATGATTAAACTATTCACG-3′ and 5′-ATGCTCTAGACACTTGAGCAGACAGCACT-3′ to amplify full-length *CG7375* cDNA in *CG7375^LL04684^* mutant; 5′-GATCGTCGATCTGGTTCTGGACAG-3′ and 5′-CCAGTGGACGAAGGCACGCTT-3′ for *α-tubulin*.

### cl-8 cells and wing disc cultures

Hh-responsive, *Drosophila* wing disc-derived clone-8 (cl-8) cells [86] were cultured at 25°C as described [33]. Effectene transfection reagent (Qiagen) was used for all transfection experiments. Cycloheximide (50 µg/ml; Sigma) was used to inhibit nascent protein synthesis in cl-8 cells. MG132 (50 mM; Sigma), ALLN (50 mM; Sigma) and lactacystin (20 mM; Boston Biochem) were used to inhibit the UPS activity. E64 (50 mM; Sigma), leupeptin (50 mM; Sigma) and NH_4_Cl (50 mM; Sigma) were used to inhibit lysosome function. In some experiments, cl-8 cells were pre-treated for 3 hours (9 hours in total) with lysosomal or UPS inhibitors prior to cycloheximide treatment for 6 hours. Third-instar larvae were dissected and incubated at 25°C for 4 hours in cl-8 cell medium supplemented with either lysosomal or UPS inhibitors before fixation.

### *In situ* hybridization, immunofluorescence staining, immunoblotting and immunoprecipitation

The coding regions of *CG13343* and *CG7375* were used to generate RNA probes for *in situ* hybridization as described previously [33]. Wing discs from third-instar larvae were fixed in 4% paraformaldehyde and labeled with the following primary antibodies: rat anti-Ci (1∶20; 2A1; gift of Robert Holmgren) [87], mouse anti-Col (1∶100; gift of Alain Vincent) [88], rabbit anti-Cul1 (1∶100; Zymed) [6], mouse anti-Dl (1∶200; C594.9B; DSHB) and rabbit anti-β-galactosidase (1∶4000; Cappel). Alexa fluor-conjugated secondary antibodies (1∶400; Invitrogen) were used. The fluorescence images were acquired with a Zeiss Axio Imager2 equipped with an ApoTome.

cl-8 cells, first-instar larvae or wing discs dissected from third-instar larvae were lysed in NP-40 buffer (1% NP-40, 150 mM NaCl and 50 mM Tris-Cl, pH 8) supplemented with protease inhibitor cocktail (Roche). Protein concentrations of the cell lysates were measured using a BCA Protein Assay (Thermo). The following antibodies were used for immunoblotting: rat anti-Ci (1∶10; 2A1), rabbit anti-Ci (1∶20000; AbN; gift of Thomas Kornberg) [89], rabbit anti-Cul1 (1∶1000; Zymed), mouse anti-Cul3 (1∶1000; BD Transduction Lab.) [13], mouse-anti-GST (1∶20000; B-14; Santa Cruz), rabbit anti-HA (1∶1000; Y-11; Santa Cruz), mouse anti-β-Tubulin (1∶6000; Covance), and mouse anti-V5 (1∶2000; Invitrogen). Note that Cul1 and Cul3 antibodies for this study have been extensively used to reveal migratory differences between neddylated and un-neddylated Cullin proteins on immunoblots [7], [22], [59]. Anti-HA-conjugated agarose (Vector Lab.) was used to immunoprecipitate dAPPBP1-HA complexes in cl-8 cells.

### *In vitro* neddylation assay

Cell-free *in vitro* neddylation assays were carried out with a NEDDylation kit according to the manufacturer's instructions (Enzo; UW0590). Typically, a 20 µl neddylation reaction includes human (supplied with the kit) or fly E1 and E2 enzymes, supplemented with 2 µl 10× NEDDylation buffer, 2 µl 10× NEDD8 (supplied with the kit), 1 µl 10× Mg-ATP (Sigma), 0.4 µl 50 mM DTT (Sigma) and 4 µl 100 U/ml IPP (NEB). For experiments shown in Figure 5C, 9.6 µl cl-8 cell lysates with or without transfected *CG13343-V5* and *dAPPBP1-HA*, and 1 µl 20× human Ubc12 (supplied with the kit) were used as E1 and E2, respectively, to neddylate Cullin proteins present in cl-8 cell lysates. For experiments shown in Figure 8C and D, 2 µl 10× human NEDD8 E1 complex (supplied with the kit) and 2 µg GST or GST-CG7375 proteins bound on Glutathione Sepharose 4B beads (GE Healthcare) were used. For experiments shown in Figure 8E, 10.6 µl cl-8 cell lysates overexpressing the fly E1 complex, and 2 µg GST-CG7375 proteins bound on GST beads were used as E1 and E2, respectively, to constitute the fly neddylation cascade *in vitro*. All immunoblotting data presented in the figures are representative of independent experiments that were performed at least three times.

## Supporting Information

Figure S1 **Expression pattern of the** ***MS1096*** **-Gal4 driver in the wing disc.** *MS1096*-GAL4-driven *mCD8-gfp* was expressed at a much higher level in the dorsal (d) compartment of the wing disc (**C**). *MS1096*-Gal4 driver alone had no effect on the expression of Dl (**A**) or CiFL (**B**). Merged image is shown in (**D**).
(TIF)

Figure S2 **Slimb as a negative regulator of CiFL stability.** Inhibition of *slimb* function by RNAi in the dorsal compartment of the wing disc (indicated by a box bracket) led to accumulation of CiFL protein (**A**) and expansion of *dpp-lacZ* activity (**B**). Similarly, knockdown of *slimb* expression cell-autonomously stabilized CiFL in an anterior clone (**D–F**, arrowhead), but was incapable of inducing *de novo* Ci expression in a posterior clone (**D–F**, arrow). Note that *ci* transcript is not expressed in posterior cells.
(TIF)

Figure S3 **Expression patterns of** ***CG13343*** **and** ***CG7375*** **in the wing disc.** Endogenous *CG13343* (**B**) and *CG7375* transcripts (**E**) were detected by *in situ* hybridization in wildtype (WT) wing discs using antisense RNA probes specific to *CG13343* and *CG7375*, respectively. Sense RNA probes (**A** and **D**) were used as the negative control. Ectopic expression of *CG13343* (**C**) and *CG7375* (**F**) was detected in the dorsal compartment of the wing disc (indicated by a box bracket) from *ap*-Gal4 driven EP[G8197] and EY[22840] flies, respectively. Note that the UAS-containing *P*-elements in EP[G8107] and EY[22804] are inserted on the 5′ UTR of *CG13343* and *CG7375*, respectively. Elevated *CG13343* or *CG7375* expression in the wing dics was not sufficient to disrupt adult wing development (data not shown), presumably due to a limited amount of Cul proteins or NEDD8 modifier in the neddylation pathway.
(TIF)

Figure S4 **The effect of** ***CG13343*** **and** ***CG7375*** **on the amounts of CiFL and Ci75 in wing discs.** (**A**) Lysates extracted from wildtype (lane 1) or *CG13343* RNAi overexpressing wing discs (lane 2) were immunoblotted (WB) with a Ci antibody (AbN), which recognizes both CiFL (ie. Ci155) and Ci75 [89]. Overexpression of *CG13343* RNAi led to a significant accumulation of CiFL. However, the amount of Ci75 was not obviously changed. β-Tubulin was used as the loading control. (**B**) Lysates extracted from wildtype (lane 1) or *CG7375* RNAi overexpressing βwing discs (lane 2) were immunoblotted with a Ci antibody (AbN). Overexpression of *CG7375* RNAi resulted in a significant accumulation of CiFL. However, the amount of Ci75 was slightly reduced.
(TIF)

Figure S5 **Reduced expression of** ***CG13343*** **and** ***CG7375*** **transcripts by RNAi and in loss-of-function alleles.** (**A** and **B**) The levels of *CG13343* (**A**) and *CG7375* mRNAs (**B**) in wing discs overexpressing RNAi transgenes were evaluated by semi-quantitative RT-PCR. PCR products were quantified by Image J densitometry. RNAi overexpression resulted in significant reduction of the expression of *CG13343* (70% reduction) and *CG7375* (90% reduction) in wing discs. In contrast, the expression of *CG11020*, which is an off-target of the *CG13343* RNAi transgene, did not change. *α-tubulin* was used as the internal control. (**C** and **D**) The levels of full-length transcripts of *CG13343* (**C**) and *CG7375* (**D**) in first-instar larvae were examined by RT-PCR. Full-length transcripts of *CG13343* (**C**) and *CG7375* (**D**) were not detected in *CG13343^SH2028^* and *CG7375^LL04684^* homozygous mutants, respectively. *a-tubulin* was used as the internal control.
(TIF)

Figure S6 **ClustalX alignment of CG13343 protein and its Uba3 orthologs in** ***Homo sapiens*** **(Hs),** ***Mus musculus*** **(Mm) and** ***Schizosaccharomyces pombe*** **(Sp).** Sequences used are Dm NP_610913.1, Hs NP_003959.3, Mm NP_ 035796.1 and Sp NP_ 592940.1. The UBA/THIF-type NAD/FAD binding domain (IPR000594) is shaded in yellow. The ubiquitin-activating enzyme repeat (IPR000127) is shaded in blue. The Nedd8 specificity determination residue is shaded in grey. The catalytic cysteine residue of E1-activating enzyme is shown in green. Purple shade marks the E2 binding domain (IPR014929).
(TIF)

Figure S7 **ClustalX alignment of CG7375 protein and its Ubc12 orthologs in** ***Homo sapiens*** **(Hs),** ***Mus musculus*** **(Mm) and** ***Schizosaccharomyces pombe*** **(Sp).** Sequences used are Dm NP_648187.1, Hs NP_003960.1, Mm NP_663553.1 and Sp NP_588256.1. The ubiquitin-conjugating enzyme E2 activity core (IPR000608) is shaded in blue. The N-terminal E1 binding motif specific for neddylation [62] and the E2-conjugating enzyme catalytic cysteine residue are shaded in yellow and green, respectively. The N-terminal E1 binding motif was deleted in GST-CG7375DN (amino acids 2–23).
(TIF)

Figure S8 **Reduced** ***dNEDD8*** **expression regulates Cul stabilization to elicite a full spectrum of Hh signaling responses.** Hypomorphic *dNEDD8^AN015^* somatic clones (negatively marked by nGFP in **B**, **E** and **H**) were induced in wing discs. Cul1 protein (**A**) was stabilized in *dNEDD8^AN015^* clones located at the anterior (arrowheads) and posterior (arrow) compartments of the wing disc. However, ectopic CiFL (**D**) and Col (**G**) were induced only in anterior clones (arrowheads).
(TIF)

Figure S9 **A model illustrating that dUba3 and dUbc12 control the stability and activity of Cul1 and Cul3 to regulate a full spectrum of Hh signaling.**
(TIF)

Table S1 **Targeted** ***in vivo*** **RNAi screen to identify the UPS regulators in Hh signaling.**
(PDF)
