# Supplementary figures and images for: In Vivo RNAi Screen Reveals Neddylation Genes as Novel Regulators of Hedgehog Signaling
Source: PLoS One. 2011 Sep 8;6(9):e24168. doi: 10.1371/journal.pone.0024168 (PMC3169580; doi:10.1371/journal.pone.0024168)

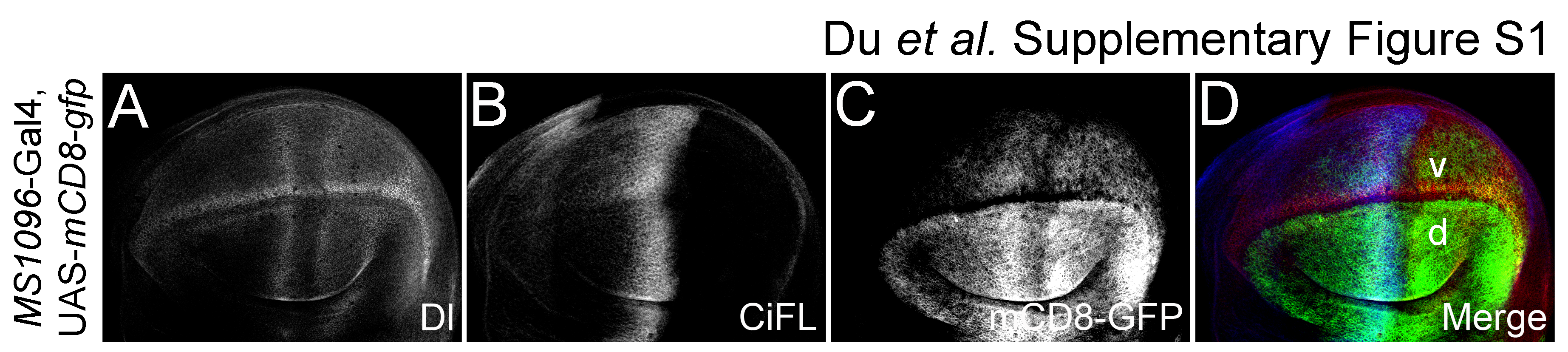

Supplement: Figure S1 — Expression pattern of the MS1096 -Gal4 driver in the wing disc. MS1096-GAL4-driven mCD8-gfp was expressed at a much higher level in the dorsal (d) compartment of the wing disc (C). MS1096-Gal4 driver alone had no effect on the expression of Dl (A) or CiFL (B). Merged image is shown in (D). (TIF) [file pone.0024168.s001.tif]

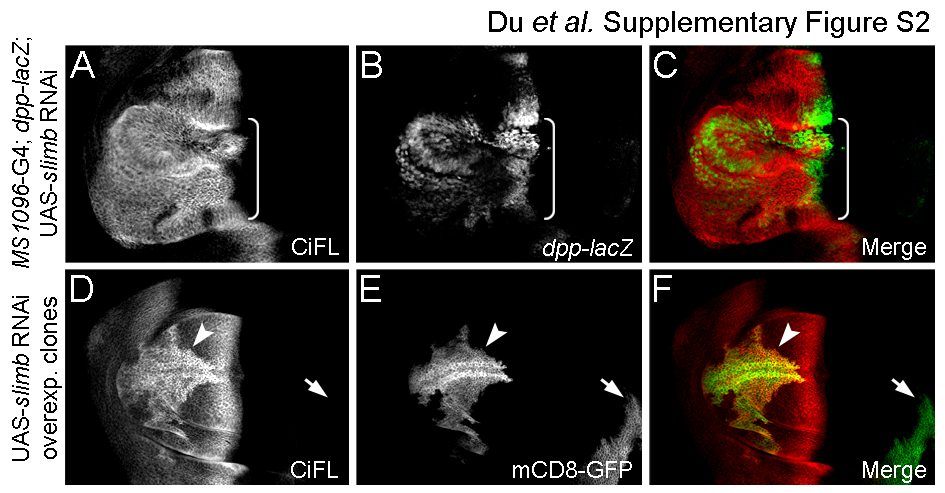

Supplement: Figure S2 — Slimb as a negative regulator of CiFL stability. Inhibition of slimb function by RNAi in the dorsal compartment of the wing disc (indicated by a box bracket) led to accumulation of CiFL protein (A) and expansion of dpp-lacZ activity (B). Similarly, knockdown of slimb expression cell-autonomously stabilized CiFL in an anterior clone (D–F, arrowhead), but was incapable of inducing de novo Ci expression in a posterior clone (D–F, arrow). Note that ci transcript is not expressed in posterior cells. (TIF) [file pone.0024168.s002.tif]

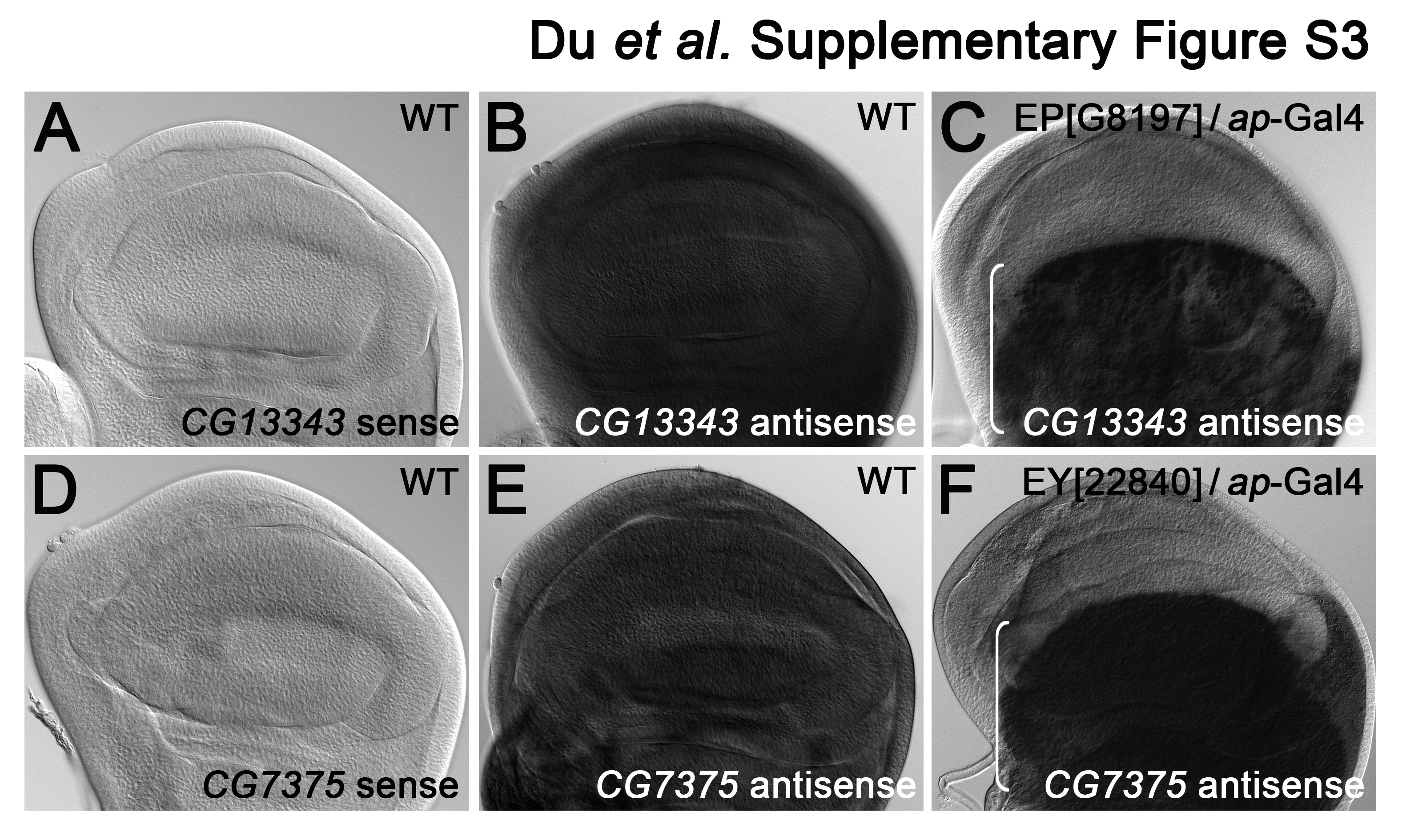

Supplement: Figure S3 — Expression patterns of CG13343 and CG7375 in the wing disc. Endogenous CG13343 (B) and CG7375 transcripts (E) were detected by in situ hybridization in wildtype (WT) wing discs using antisense RNA probes specific to CG13343 and CG7375, respectively. Sense RNA probes (A and D) were used as the negative control. Ectopic expression of CG13343 (C) and CG7375 (F) was detected in the dorsal compartment of the wing disc (indicated by a box bracket) from ap-Gal4 driven EP[G8197] and EY[22840] flies, respectively. Note that the UAS-containing P-elements in EP[G8107] and EY[22804] are inserted on the 5′ UTR of CG13343 and CG7375, respectively. Elevated CG13343 or CG7375 expression in the wing dics was not sufficient to disrupt adult wing development (data not shown), presumably due to a limited amount of Cul proteins or NEDD8 modifier in the neddylation pathway. (TIF) [file pone.0024168.s003.tif]

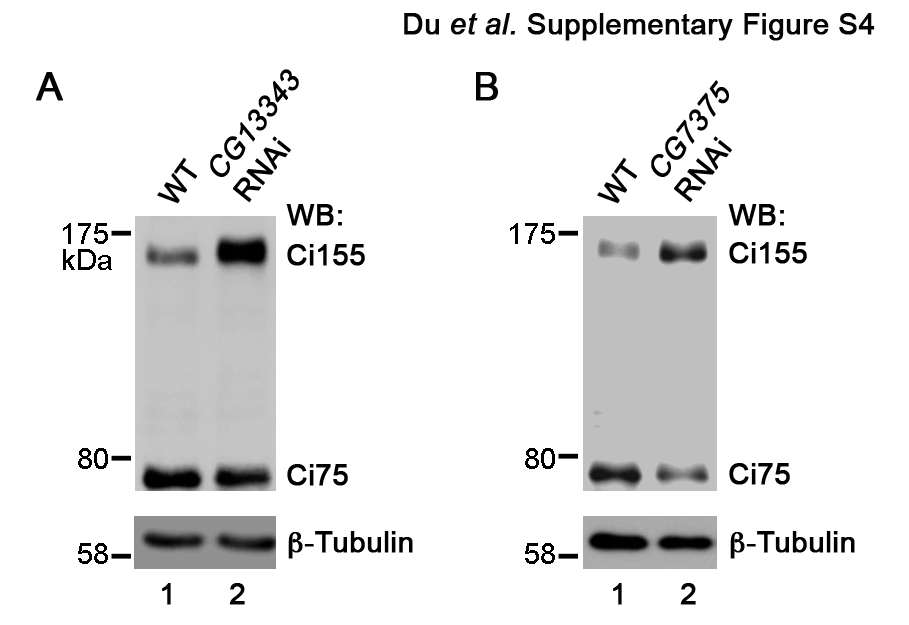

Supplement: Figure S4 — The effect of CG13343 and CG7375 on the amounts of CiFL and Ci75 in wing discs. (A) Lysates extracted from wildtype (lane 1) or CG13343 RNAi overexpressing wing discs (lane 2) were immunoblotted (WB) with a Ci antibody (AbN), which recognizes both CiFL (ie. Ci155) and Ci75 [89]. Overexpression of CG13343 RNAi led to a significant accumulation of CiFL. However, the amount of Ci75 was not obviously changed. β-Tubulin was used as the loading control. (B) Lysates extracted from wildtype (lane 1) or CG7375 RNAi overexpressing βwing discs (lane 2) were immunoblotted with a Ci antibody (AbN). Overexpression of CG7375 RNAi resulted in a significant accumulation of CiFL. However, the amount of Ci75 was slightly reduced. (TIF) [file pone.0024168.s004.tif]

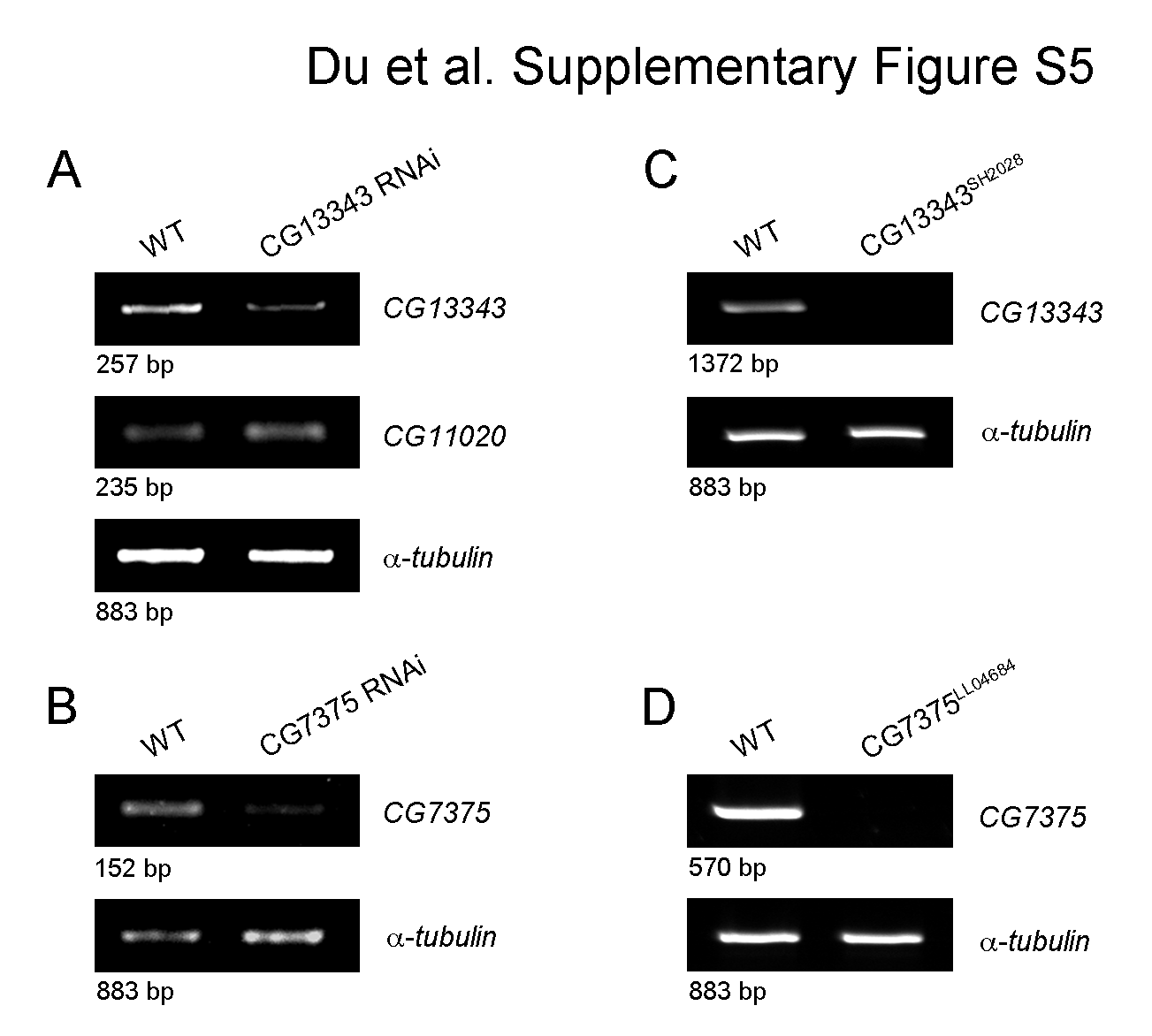

Supplement: Figure S5 — Reduced expression of CG13343 and CG7375 transcripts by RNAi and in loss-of-function alleles. (A and B) The levels of CG13343 (A) and CG7375 mRNAs (B) in wing discs overexpressing RNAi transgenes were evaluated by semi-quantitative RT-PCR. PCR products were quantified by Image J densitometry. RNAi overexpression resulted in significant reduction of the expression of CG13343 (70% reduction) and CG7375 (90% reduction) in wing discs. In contrast, the expression of CG11020, which is an off-target of the CG13343 RNAi transgene, did not change. α-tubulin was used as the internal control. (C and D) The levels of full-length transcripts of CG13343 (C) and CG7375 (D) in first-instar larvae were examined by RT-PCR. Full-length transcripts of CG13343 (C) and CG7375 (D) were not detected in CG13343SH2028 and CG7375LL04684 homozygous mutants, respectively. a-tubulin was used as the internal control. (TIF) [file pone.0024168.s005.tif]

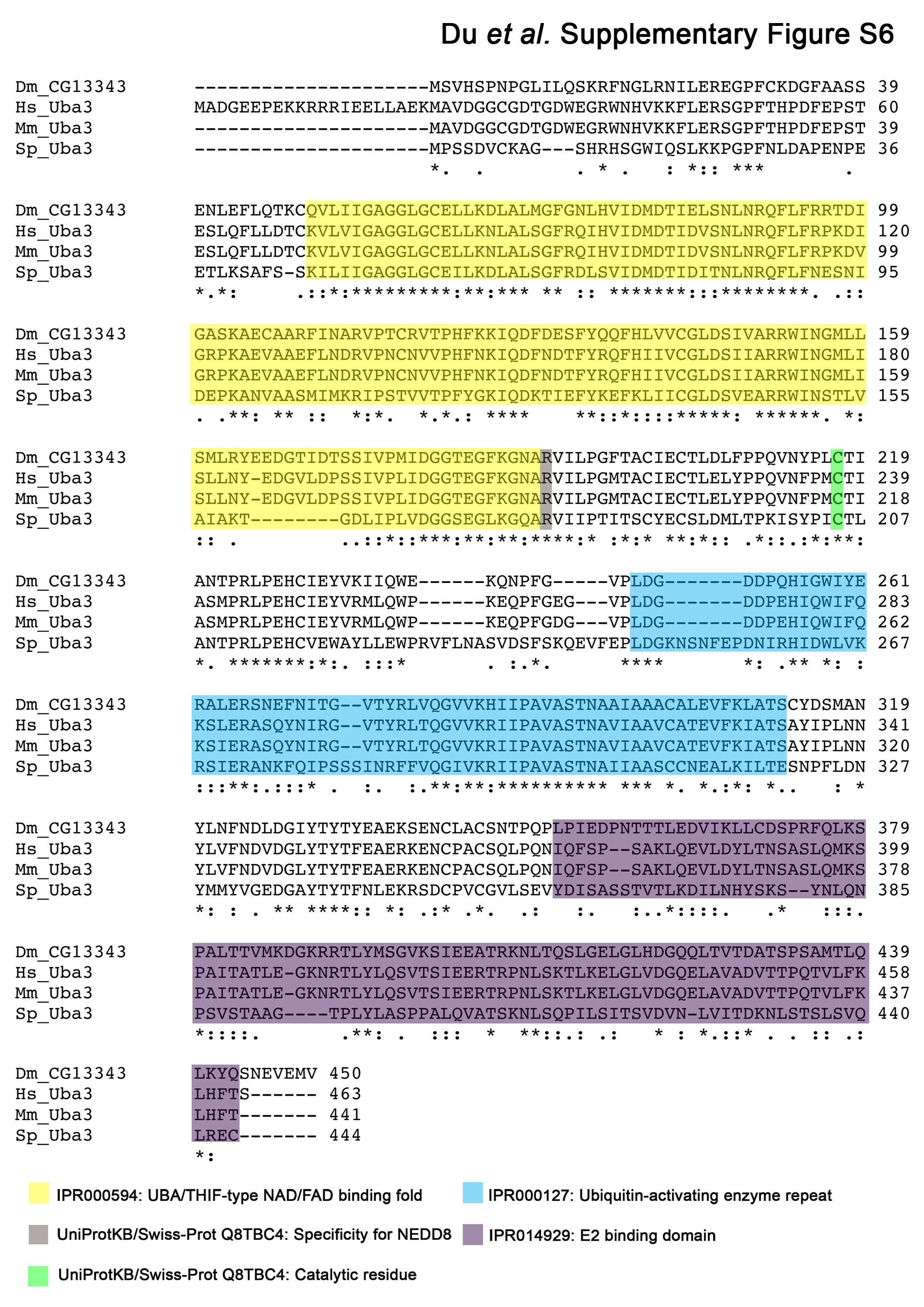

Supplement: Figure S6 — ClustalX alignment of CG13343 protein and its Uba3 orthologs in Homo sapiens (Hs), Mus musculus (Mm) and Schizosaccharomyces pombe (Sp). Sequences used are Dm NP_610913.1, Hs NP_003959.3, Mm NP_ 035796.1 and Sp NP_ 592940.1. The UBA/THIF-type NAD/FAD binding domain (IPR000594) is shaded in yellow. The ubiquitin-activating enzyme repeat (IPR000127) is shaded in blue. The Nedd8 specificity determination residue is shaded in grey. The catalytic cysteine residue of E1-activating enzyme is shown in green. Purple shade marks the E2 binding domain (IPR014929). (TIF) [file pone.0024168.s006.tif]

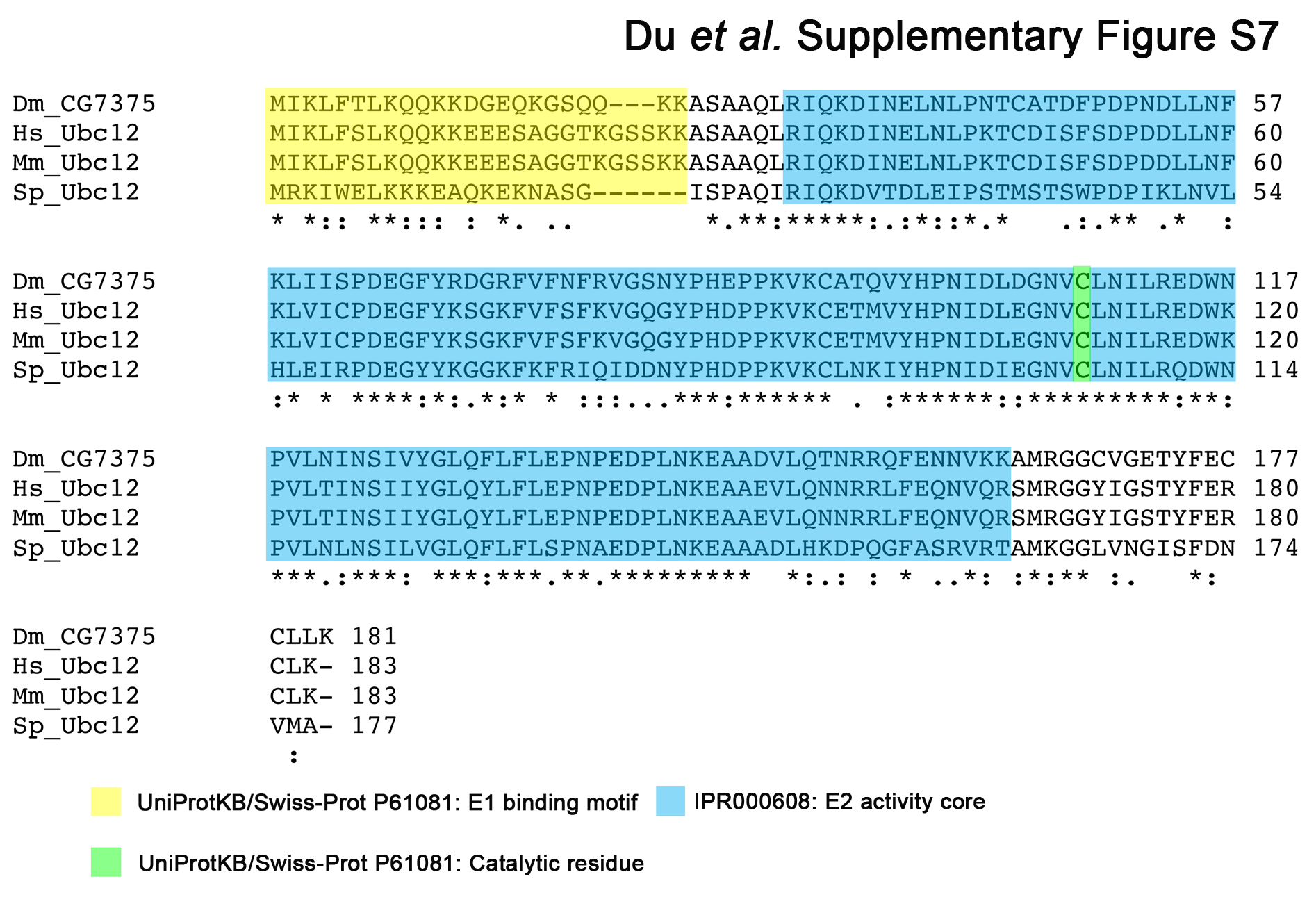

Supplement: Figure S7 — ClustalX alignment of CG7375 protein and its Ubc12 orthologs in Homo sapiens (Hs), Mus musculus (Mm) and Schizosaccharomyces pombe (Sp). Sequences used are Dm NP_648187.1, Hs NP_003960.1, Mm NP_663553.1 and Sp NP_588256.1. The ubiquitin-conjugating enzyme E2 activity core (IPR000608) is shaded in blue. The N-terminal E1 binding motif specific for neddylation [62] and the E2-conjugating enzyme catalytic cysteine residue are shaded in yellow and green, respectively. The N-terminal E1 binding motif was deleted in GST-CG7375DN (amino acids 2–23). (TIF) [file pone.0024168.s007.tif]

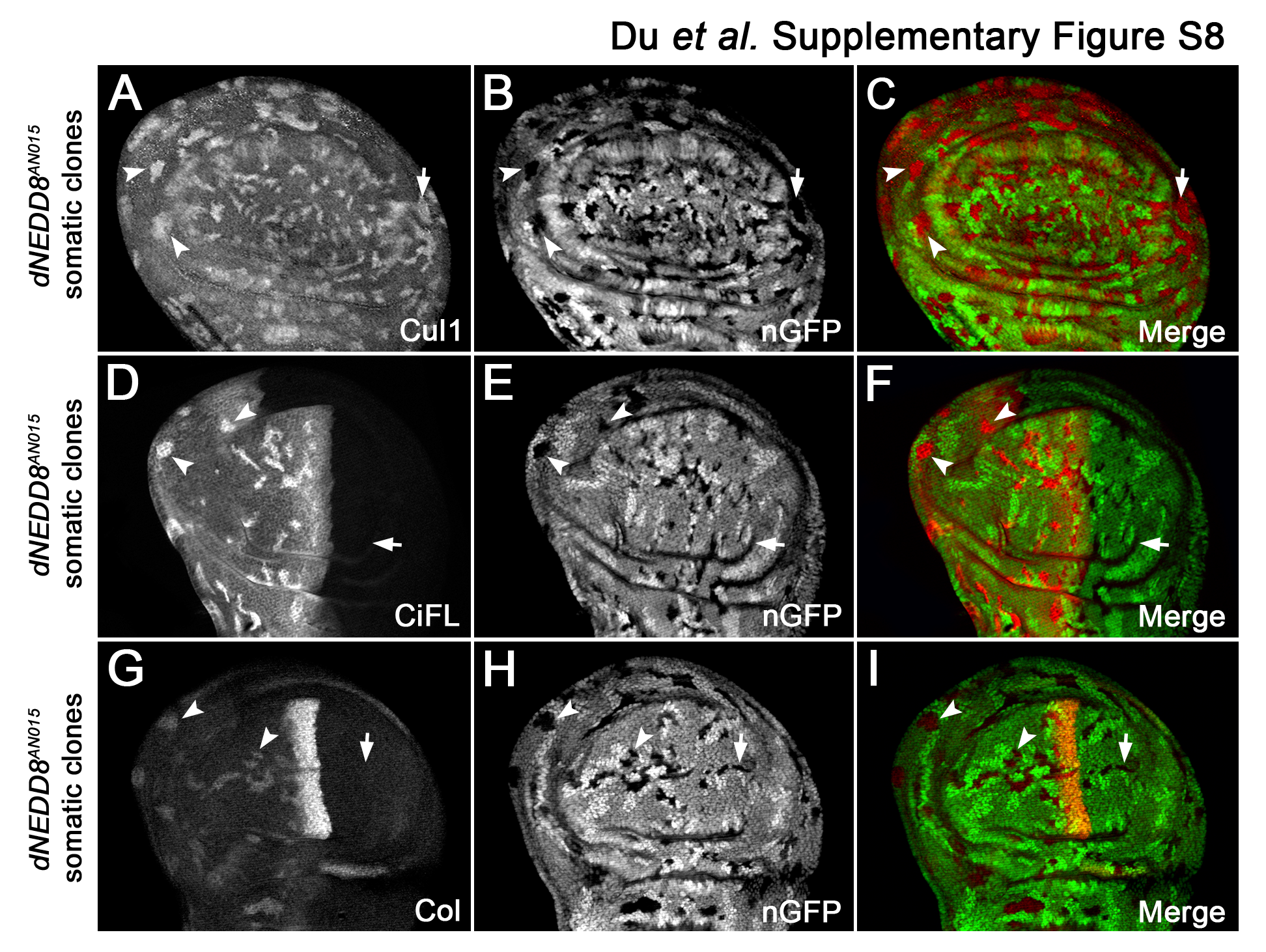

Supplement: Figure S8 — Reduced dNEDD8 expression regulates Cul stabilization to elicite a full spectrum of Hh signaling responses. Hypomorphic dNEDD8AN015 somatic clones (negatively marked by nGFP in B, E and H) were induced in wing discs. Cul1 protein (A) was stabilized in dNEDD8AN015 clones located at the anterior (arrowheads) and posterior (arrow) compartments of the wing disc. However, ectopic CiFL (D) and Col (G) were induced only in anterior clones (arrowheads). (TIF) [file pone.0024168.s008.tif]

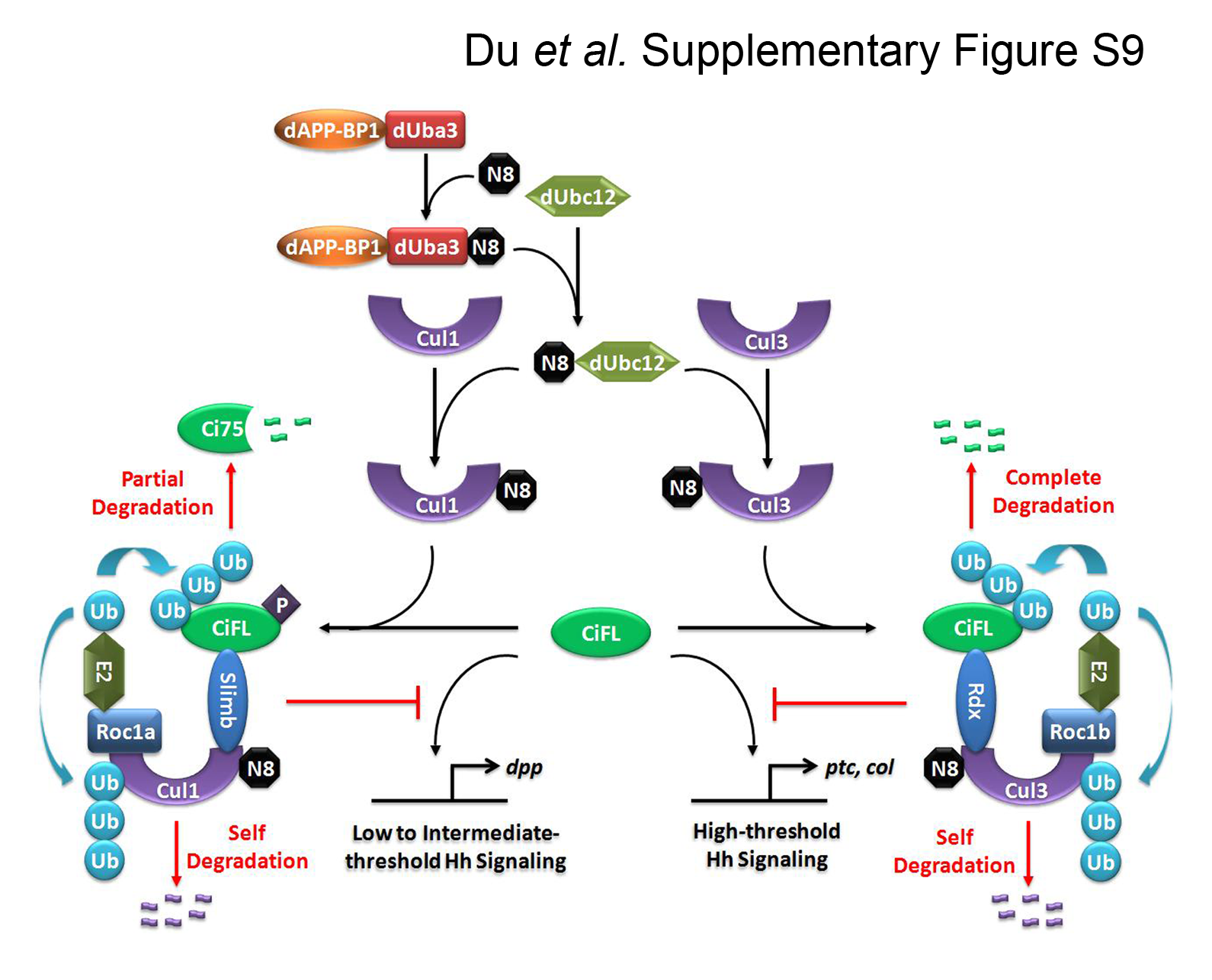

Supplement: Figure S9 — A model illustrating that dUba3 and dUbc12 control the stability and activity of Cul1 and Cul3 to regulate a full spectrum of Hh signaling. (TIF) [file pone.0024168.s009.tif]
