# Supplementary material for: In Vivo RNAi Screen Reveals Neddylation Genes as Novel Regulators of Hedgehog Signaling
Source: PLoS One. 2011 Sep 8;6(9):e24168. doi: 10.1371/journal.pone.0024168 (PMC3169580; doi:10.1371/journal.pone.0024168)
Supplement: Table S1 — Targeted in vivo RNAi screen to identify the UPS regulators in Hh signaling. (PDF) [file pone.0024168.s010.pdf]

E1 genes IPR000594

CG number	Gene symbol	RNAi line	MS1096 phenotype	Ci staining
CG12276	*Aos1*	V18528*	Severely damaged	No phenotype
	Aos1	V47256	Severely damaged	No phenotype
CG13090		V43558	No phenotype	
CG13343		V17137	Blistered	Up-regulated
		V105141	Blistered	Up-regulated
CG1749		V32937	Larval lethal	
CG1782	*Uba1*	N1782R-2**	Pupal lethal	Up-regulated
CG5489	*Atg7*	V27432	No phenotype	
	Atg7	V45558	No phenotype	
CG7528	*Uba2*	N7528R-1	Severely damaged	Disc malformed
	Uba2	N7528R-2	Severely damaged	Disc malformed
CG7828	*APPBP1*	V7728	No phenotype	
	**8 genes**	**12 RNAi lines**		

E2 genes IPR000608

CG number	Gene symbol	RNAi line	MS1096 phenotype	Ci staining
CG10254		V15992	No phenotype	
CG10536	*cbx*	V16078	No phenotype	
	cbx	V101755	No phenotype	
CG10640	*Uev1A*	V32267	No phenotype	
	Uev1A	V107465	Bent	No phenotype
CG10682	*vih*	V27306	No phenotype	
		V107720	No phenotype	
CG10723	*Kua*	V30305	No phenotype	
	Kua	V101360	Blistered	No phenotype
CG10862		V31372	No phenotype	
		V101113	Bent	No phenotype
CG12799	*Ubc84D*	V20260	No phenotype	
	Ubc84D	V106363	No phenotype	
CG14739		V18358	No phenotype	
		V105594	No phenotype	
CG15437	*morgue*	N15437R-1	No phenotype	
E2/F-box		V11090	No phenotype	
CG16894		V10067	No phenotype	
CG17030		V32827	No phenotype	
		V32828	No phenotype	
CG18319	*ben*	V9413	Pupal lethal	Disc malformed
CG2013	*UbcD6*	V23230	Pupal lethal	Disc malformed
	UbcD6	V46927	Pupal lethal	Disc malformed
CG2257	*Ubc-E2H*	V33509	Bent	No phenotype
	Ubc-E2H	V33510	Bent	No phenotype
CG2574		V40173	No phenotype	
		V105725	No phenotype	

CG2924		V104482	No phenotype	
CG3018	*lwr*	V33685	Pupal lethal	Disc malformed
	lwr	V104007	Pupal lethal	Disc malformed
CG3473		V26201	No phenotype	
		V104207	Bent	No phenotype
CG40045		V109167	No phenotype	
CG4443	*crl*	V34111	No phenotype	
	crl	V104440	No phenotype	
CG4502		V34855	Bent	No phenotype
		V34858	No phenotype	
CG5440		V49030	No phenotype	
		V100099	No phenotype	
CG5788	*UbcD10*	V27515	No phenotype	
	UbcD10	V48146	No phenotype	
	UbcD10	V100570	Pupal lethal	No phenotype
CG5823		V8301	No phenotype	
		V33260	No phenotype	
CG6303	*Bruce*	V48309	No phenotype	
	Bruce	V107620	No phenotype	
CG6720	*UbcD2*	V31158	No phenotype	
CG7220		V34199	No phenotype	
		V104478	No phenotype	
CG7375		V35219	Severely damaged	Up-regulated
		V100761	Severely damaged	Up-regulated
CG7425	*eff*	V26011	Pupal lethal	Disc malformed
	eff	V105731	Pupal lethal	Disc malformed
CG7656		V26880	No phenotype	
		V100791	No phenotype	
CG8188		V103362	No phenotype	
CG8284	*UbcD4*	V35873	No phenotype	
	UbcD4	V106600	No phenotype	
CG9602		V29499	No phenotype	
		V102641	No phenotype	
CG9712	*TSG101*	V23944	Pupal lethal	Disc malformed
	**34 genes**	**61 RNAi lines**		

E3 genes IPR000569 HECT domain

CG number	Gene symbol	RNAi line	MS1096 phenotype	Ci staining
CG11734	*HERC2*	N11734R-1	No phenotype	
	HERC2	N11734R-3	No phenotype	
CG17735		N17735R-2	No phenotype	
		V108546	Bent	No phenotype
CG3099		N3099R-2	No phenotype	
		N3099R-3	No phenotype	
CG3356		N3356R-1	Blistered	No phenotype

		V101229	No phenotype
CG4238		V41982	No phenotype
CG4244	*Su(dx)*	N4244R-1	No phenotype
		N4244R-2	No phenotype
CG4943	*lack*	N4943R-1	No phenotype
		N4943R-2	No phenotype
CG5087		N5087R-2	No phenotype
		N5087R-3	No phenotype
CG5604		V27467	No phenotype
CG6190	*Ube3a*	N6190R-1	No phenotype
	Ube3a	N6190R-3	No phenotype
CG7555	*Nedd4*	N7555R-1	No phenotype
CG8184		N8184R-1	No phenotype
		N8184R-6	No phenotype
CG9153		V37220	No phenotype
		V37221	No phenotype
CG9484	*hyd*	N9484R-1	No phenotype
	hyd	N9484R-2	No phenotype
	**14 genes**	**25 RNAi lines**	

E3 genes IPR001841 RING domain

CG number	Gene symbol	RNAi line	MS1096 phenotype	Ci staining
CG10144		V18019	No phenotype	
		V105952	No phenotype	
CG10263		N/A***		
CG10277		V7518	No phenotype	
CG10542	*Bre1*	V15620	Bent	No phenotype
	Bre1	V108206	Bent	No phenotype
CG10916		V31379	No phenotype	
		V107518	No phenotype	
CG10961	*Traf6*	V16125	No phenotype	
	Traf6	V16126	No phenotype	
CG10981		N10981R-3	No phenotype	
		N10981R-4	No phenotype	
CG11281	*snky*	V5246	No phenotype	
		V5247	No phenotype	
CG11321		N11321R-3	No phenotype	
CG1134		N1134R-2	No phenotype	
CG11360		N11360R-2	No phenotype	
		N11360R-4	No phenotype	
CG11414		N11414R-1	No phenotype	
		N11414R-3	No phenotype	
CG11534		N11534R-1	No phenotype	
		N11534R-3	No phenotype	
CG11982		V38623	Blistered	No phenotype
		V105651	No phenotype	

CG11988	*neur*	V11988R-1	No phenotype	
	neur	V11988R-3	No phenotype	
CG12099		V18734	No phenotype	
CG12200		N12200R-1	No phenotype	
		N12200R-3	No phenotype	
CG12218	*mei-P26*	V31695	Pupal lethal	Disc malformed
	mei-P26	V101060	No phenotype	
CG12284	*th*	N12284R-2	No phenotype	
CG12362		N12362R-1	No phenotype	
		N12362R-3	No phenotype	
CG12477		V31944	No phenotype	
		V102882	No phenotype	
CG12489	*dnr1*	N12489R-1	No phenotype	
CG13025		N13025R-1	No phenotype	
		N13025R-2	No phenotype	
CG13030	*sinah*	N13030R-2	No phenotype	
		N13030R-3	No phenotype	
CG13344		N13344R-1	No phenotype	
CG13481		V17205	No phenotype	
		V103527	No phenotype	
CG13605		V105112	No phenotype	
CG13835		N13835R-2	No phenotype	
		N13835R-4	No phenotype	
CG14306		V106368	No phenotype	
CG14435		N14435R-1	Blistered	No phenotype
		N14435R-4	Blistered	No phenotype
CG14983		V32341	No phenotype	
		V104309	No phenotype	
CG15011		N15011R-3	No phenotype	
CG15104	*Topors*	N15104R-2	No phenotype	
	Topors	N15104R-3	No phenotype	
CG15105	*abba*	V19290	No phenotype	
	abba	V107067	No phenotype	
CG15150	*elfless*	N15150R-1	No phenotype	
CG15439		V19490	Pupal lethal	Disc malformed
CG15814		V30429	Bent	No phenotype
		V100725	Bent	No phenotype
CG16807	*roq*	V23843	No phenotype	
CG16947		V108103	No phenotype	
CG16982	*Roc1a*	V32399	Pupal lethal	Up-regulated
	Roc1a	V106315	Pupal lethal	Up-regulated
CG16988	*Roc1b*	V32797	Pupal lethal	Up-regulated
	Roc1b	V32798	Pupal lethal	Up-regulated
CG17019		N17019R-3	No phenotype	
		N17019R-6	No phenotype	
CG17033	*elgi*	N17033R-2	No phenotype	
	elgi	N17033R-3	No phenotype	
CG17048		V8780	No phenotype	
		V101890	No phenotype	

CG17260		N17260R-1	No phenotype	
		N17260R-2	No phenotype	
CG17329		V19171	Bent	No phenotype
CG17492	*mib2*	N17492R-1	No phenotype	
	mib2	N17492R-3	No phenotype	
CG17721		V6036	No phenotype	
CG18028	*lt*	N/A		
CG1815		N1815R-1	No phenotype	
		V107321	Bent	No phenotype
CG1909		V20745	No phenotype	
CG1937	*sip3*	V6870	No phenotype	
	sip3	V107060	No phenotype	
CG2304	*Trc8*	N2304R-2	No phenotype	
	Trc8	N2304R-3	No phenotype	
CG2617		N2617R-1	No phenotype	
CG2679	*gol*	N2679R-3	No phenotype	
CG2681		N2681R-2	No phenotype	
CG2682	*d4*	N2682R-1	No phenotype	
CG2709		N2709R-2	No phenotype	
		N2709R-3	No phenotype	
CG2926		N2926R-2	No phenotype	
CG3093	*dor*	V33733	Bent	No phenotype
	dor	V107053	Bent	No phenotype
CG31053		N31053R-1	No phenotype	
		N31053R-3	No phenotype	
CG31392		V25756	No phenotype	
		V101163	No phenotype	
CG31716		N31716R-1	No phenotype	
		N31716R-2	No phenotype	
CG31721	*Trim9*	V21405	Bent	No phenotype
	Trim9	V100767	Bent	No phenotype
CG31807		N31807R-2	No phenotype	
		N31807R-3	No phenotype	
CG32096	*rols*	N/A		
CG32210	*l(3)76BDr*	N9268R-1	No phenotype	
	l(3)76BDr	N9268R-3	No phenotype	
CG3231	*snama*	N3231R-1	Pupal lethal	Disc malformed
CG32350		V24732	No phenotype	
		V107420	No phenotype	
CG32369		V45129	No phenotype	
		V103923	No phenotype	
CG3241	*msl-2*	V29356	Bent only in males	No phenotype
	msl-2	V102386	Bent only in males	No phenotype
CG32486		V41907	Severely damaged	No phenotype
		V41908	Severely damaged	No phenotype
CG32581		V49727	Blistered	No phenotype
		V49728	Blistered	No phenotype
CG32592	*hiw*	V28163	Bent	No phenotype
	hiw	V36085	Bent	No phenotype

CG32847		V48422	No phenotype	
		V104294	No phenotype	
CG32850		N/A		
CG33144		V17081	Blistered	No phenotype
		V108583	No phenotype	
CG33552		V47843	No phenotype	
		V47844	No phenotype	
CG34289		N/A		
CG34308		N/A		
CG34440	*lmg*	N/A		
CG3639	*pex12*	N3639R-1	No phenotype	
CG3647	*stc*	V47973	No phenotype	
	stc	V47974	No phenotype	
CG3886	*Psc*	N3886R-1	Pupal lethal	Disc malformed
	Psc	V30587	Pupal lethal	Disc malformed
CG3905	*Su(z)2*	V50368	Pupal lethal	Disc malformed
	Su(z)2	V100096	Severely damaged	Disc malformed
CG3929	*dx*	N3929R-1	No phenotype	
	dx	N3929R-2	No phenotype	
CG4030		V26367	No phenotype	
		V26368	No phenotype	
CG4195	*l(3)73Ah*	N4195R-1	No phenotype	
	l(3)73Ah	N4195R-4	No phenotype	
CG42593		V5468	Pupal lethal	Disc malformed
		V45166	No phenotype	
		V22901	Severely damaged	Disc malformed
		V106993	Pupal lethal	Disc malformed
CG4325		V102993	No phenotype	
CG4620	*unk*	V4267	No phenotype	
	unk	V104665	Bent	No phenotype
CG4813		N4813R-1	No phenotype	
		N4813R-3	No phenotype	
CG4909	*POSH*	N4909R-1	No phenotype	
	POSH	N4909R-3	No phenotype	
CG4973		N4973R-1	No phenotype	
		N4973R-4	No phenotype	
CG4976	*Mes-4*	V10836	No phenotype	
	Mes-4	V10837	No phenotype	
CG5071		N5071R-1	No phenotype	
		N5071R-2	No phenotype	
CG5140	*nopo*	V22013	No phenotype	
	nopo	V104477	No phenotype	
CG5206	*bon*	V44283	Blistered	No phenotype
		V101737	Pupal lethal	No phenotype
CG5334		N5334R-1	No phenotype	
		N5334R-3	No phenotype	
CG5347		N5347R-1	No phenotype	
		N5347R-3	No phenotype	
CG5382		V101394	No phenotype	

CG5555		V35012	Pupal lethal	No phenotype
		V110162	Pupal lethal	No phenotype
CG5591		V22170	No phenotype	
		V22171	No phenotype	
CG5595	*Sce*	V27465	Pupal lethal	Disc malformed
	Sce	V106328	Pupal lethal	Disc malformed
CG5659	*ari-1*	V35029	No phenotype	
	ari-1	V39591	No phenotype	
CG5709	*ari-2*	N5709R-1	No phenotype	
CG5841	*mib1*	V27525	Pupal lethal	No phenotype
	mib1	V27526	Pupal lethal	No phenotype
CG6613		V42065	No phenotype	
		V42066	No phenotype	
CG6688		N6688R-3	No phenotype	
CG6752		N6752R-1	No phenotype	
CG6923		V26096	No phenotype	
		V108095	No phenotype	
CG7037	*Cbl*	N7037R-1	No phenotype	
	Cbl	N7037R-2	No phenotype	
CG7081	*pex2*	N7081R-2	No phenotype	
CG7184	*Mkrn1*	V34373	No phenotype	
	Mkrn1	V101694	No phenotype	
CG7376		V35222	No phenotype	
CG7614	*Mat1*	V12575	Blistered	No phenotype
	Mat1	V104780	Bent	No phenotype
CG7694		N7694R-1	No phenotype	
		N7694R-2	No phenotype	
CG7864	*pex10*	V46613	No phenotype	
CG8103	*Mi-2*	V10766	Pupal lethal	Disc malformed
	Mi-2	V107204	Pupal lethal	Disc malformed
CG8141		V37349	No phenotype	
		V101087	No phenotype	
CG8293	*Iap2*	N8293R-1	Severely damaged	Disc malformed
	Iap2	N8293R-2	Severely damaged	Disc malformed
CG8419		V24097	No phenotype	
		V107626	No phenotype	
CG8651	*trx*	V37715	Blistered	Disc malformed
	trx	V108122	Pupal lethal	Disc malformed
CG8786		N8786R-1	No phenotype	
		N8786R-4	No phenotype	
CG8910		N8910R-1	No phenotype	
		N8910R-3	No phenotype	
CG8974		N8974R-2	Severely damaged	Disc malformed
		V5572	Pupal lethal	Disc malformed
CG8998	*Roc2*	V28102	Bent	No phenotype
	Roc2	V100629	Bent	No phenotype
CG9014		N9014R-1	No phenotype	
		N9014R-2	No phenotype	
CG9086		N9086R-3	No phenotype	

CG9381	*mura*	N/A		
CG9576		N9576R-1	No phenotype	
		N9576R-3	No phenotype	
CG9941		V29596	No phenotype	
		V105826	No phenotype	
CG9949	*sina*	N9949R-1	No phenotype	
	sina	N9949R-2	No phenotype	
	**134 genes**	**219 RNAi lines**		

E3 genes IPR001373 Cullin domain

CG number	Gene symbol	RNAi line	MS1096 phenotype	Ci staining
CG11261		V31478	No phenotype	
		V31479	No phenotype	
CG11861	*Cul-3*	N11861R-1	Pupal lethal	No phenotype
	Cul-3	N11861R-2	Pupal lethal	No phenotype
CG1401	*Cul-5*	N1401R-1	No phenotype	
	Cul-5	N1401R-2	No phenotype	
CG1512	*Cul-2*	N1512R-1	No phenotype	
	Cul-2	N1512R-3	No phenotype	
CG1877	*Cul-1*	N1877R-1	Pupal lethal	Up-reguated
	Cul-1	N1877R-2	Pupal lethal	Up-reguated
CG3060	*mr*	N3060R-2	Bent	No phenotype
	mr	V106986	Pupal lethal	Disc malformed
CG8711	*Cul-4*	N8711R-2	No phenotype	
	Cul-4	V105668	Pupal lethal	Disc malformed
	**7 genes**	**14 RNAi lines**		

E3 genes IPR001810 F-Box

CG number	Gene symbol	RNAi line	MS1096 phenotype	Ci staining
CG10855		V42927	No phenotype	
		V107547	No phenotype	
CG11033		V31402	No phenotype	
CG11044		N11044R-1	No phenotype	
		N11044R-2	No phenotype	
CG11866		V31583	No phenotype	
		V107678	No phenotype	
CG12402		V31932	No phenotype	
		V102835	Bent	No phenotype
CG12520		V47627	No phenotype	
		V107605	No phenotype	
CG12765		N12765R-1	No phenotype	
		N12765R-3	No phenotype	
CG13213	*fbl6*	V43002	Bent	No phenotype

	fbl6	V100758	No phenotype	
CG14937		V40819	No phenotype	
		V101001	No phenotype	
CG15010	*ago*	N15010R-2	Blistered	Disc malformed
		N15010R-3	Blistered	Disc malformed
CG15437	*morgue*	N15437R-1	No phenotype	
E2/F-box		V11090	No phenotype	
CG1839	*Fbxl4*	V15475	No phenotype	
		V15476	No phenotype	
CG2010		N2010R-1	Severely damaged	Disc malformed
		V100736	Blistered	No phenotype
CG2247		V43417	No phenotype	
CG30466		N/A		
CG31633		N31633R-2	No phenotype	
CG32085		N32085R-1	Severely damaged	Disc malformed
CG32221		V21672	No phenotype	
CG34025		N/A		
CG3412	*slmb*	N3412R-1	Pupal lethal	Up-regulated
	slmb	N3412R-3	Pupal lethal	Up-regulated
CG3428	*pall*	N3428R-1	No phenotype	
	pall	N3428R-3	No phenotype	
CG4221		V34810	No phenotype	
CG4643	*Fsn*	V26578	No phenotype	
	Fsn	V107315	No phenotype	
CG4911		N4911R-1	Bent	No phenotype
CG5003		N5003R-1	No phenotype	
		N5003R-3	Blistered	No phenotype
CG5961		N5961R-1	No phenotype	
CG6758		V43606	No phenotype	
CG7148		N7148R-1	No phenotype	
		N7148R-2	No phenotype	
CG7707		N7707R-1	No phenotype	
CG8272		N8272R-1	Blistered	No phenotype
		N8272R-2	No phenotype	
CG8873	*jet*	V45618	No phenotype	
	jet	V104399	No phenotype	
CG9003		V23481	No phenotype	
		V23482	No phenotype	
CG9144	*Fbw5*	N9144R-1	No phenotype	
	Fbw5	N9144R-2	No phenotype	
CG9316		N9316R-1	No phenotype	
CG9461	*FBX011*	V24039	No phenotype	
	FBX011	V24041	No phenotype	
CG9772		V15636	Bent	No phenotype
		V101487	Bent	No phenotype
CG9952	*ppa*	N9952R-1	No phenotype	
	ppa	N9952R-2	No phenotype	
	**37 genes**	**59 RNAi lines**		

E3 genes IPR001232 SKP1 domain

CG number	Gene symbol	RNAi line	MS1096 phenotype	Ci staining
CG11941	*skpC*	N11941R-3	Blistered	Disc malformed
	skpC	V109047	No phenotype	
CG11942	*skpE*	N11942R-3	Blistered	Disc malformed
	skpE	V109539	No phenotype	
CG12227	*skpF*	N12227R-1	Severely damaged	No phenotype
	skpF	N12227R-2	Severely damaged	No phenotype
CG12700	*skpD*	N12700R-2	Severely damaged	Disc malformed
	skpD	V49122	No phenotype	
CG15800		V19615	No phenotype	
CG16983	*skpA*	N16983R-3	No phenotype	
CG8881	*skpB*	N8881R-4	No phenotype	
CG9291	*Elongin-C*	V15302	No phenotype	
	Elongin-C	V105740	Severely damaged	Disc malformed
	**8 genes**	**13 RNAi lines**		

E3 genes IPR003613 U-Box

CG number	Gene symbol	RNAi line	MS1096 phenotype	Ci staining
CG11070		V31413	No phenotype	
		V31414	No phenotype	
CG2218		N2218R-2	No phenotype	
		N2218R-4	No phenotype	
CG5203	*CHIP*	N5203R-2	No phenotype	
	CHIP	N5203R-3	No phenotype	
CG5519	*Prp19*	N5519R-1	Pupal lethal	Disc malformed
	Prp19	V108575	Pupal lethal	Disc malformed
CG9934		V36464	No phenotype	
	**5 genes**	**9 RNAi lines**		

E3 genes IPR003126 Zinc finger, N-recognin

CG number	Gene symbol	RNAi line	MS1096 phenotype	Ci staining
CG14472	*poe*	N14472R-1	No phenotype	
	poe	N14472R-3	No phenotype	
CG15141		N15141R-1	No phenotype	
		N15141R-4	No phenotype	
	**2 genes**	**4 RNAi lines**		

*: RNAi lines with their names starting with "V" were obtained from the VDRC.
**: RNAi lines with their names starting with "N" were obtained from the NIG-Fly.
***: RNAi lines were not available for a total of 10 genes.
